# IL-27 Receptor Signalling Restricts the Formation of Pathogenic, Terminally Differentiated Th1 Cells during Malaria Infection by Repressing IL-12 Dependent Signals

**DOI:** 10.1371/journal.ppat.1003293

**Published:** 2013-04-11

**Authors:** Ana Villegas-Mendez, J. Brian de Souza, Seen-Wai Lavelle, Emily Gwyer Findlay, Tovah N. Shaw, Nico van Rooijen, Christiaan J. Saris, Christopher A. Hunter, Eleanor M. Riley, Kevin N. Couper

**Affiliations:** 1 Department of Immunology and Infection, Faculty of Infectious and Tropical Diseases, London School of Hygiene and Tropical Medicine, London, United Kingdom; 2 Department of Immunology and Molecular Pathology, University College London Medical School, London, United Kingdom; 3 Department of Molecular Cell Biology, VU Medical Center, Amsterdam, The Netherlands; 4 Department of Inflammation Research, Amgen, Inc., Thousand Oaks, California, United States of America; 5 Department of Pathobiology, University of Pennsylvania, Philadelphia, Pennsylvania, United States of America; McGill University, Canada

## Abstract

The IL-27R, WSX-1, is required to limit IFN-γ production by effector CD4^+^ T cells in a number of different inflammatory conditions but the molecular basis of WSX-1-mediated regulation of Th1 responses *in vivo* during infection has not been investigated in detail. In this study we demonstrate that WSX-1 signalling suppresses the development of pathogenic, terminally differentiated (KLRG-1^+^) Th1 cells during malaria infection and establishes a restrictive threshold to constrain the emergent Th1 response. Importantly, we show that WSX-1 regulates cell-intrinsic responsiveness to IL-12 and IL-2, but the fate of the effector CD4^+^ T cell pool during malaria infection is controlled primarily through IL-12 dependent signals. Finally, we show that WSX-1 regulates Th1 cell terminal differentiation during malaria infection through IL-10 and Foxp3 independent mechanisms; the kinetics and magnitude of the Th1 response, and the degree of Th1 cell terminal differentiation, were comparable in WT, IL-10R1^−/−^ and IL-10^−/−^ mice and the numbers and phenotype of Foxp3^+^ cells were largely unaltered in WSX-1^−/−^ mice during infection. As expected, depletion of Foxp3^+^ cells did not enhance Th1 cell polarisation or terminal differentiation during malaria infection. Our results significantly expand our understanding of how IL-27 regulates Th1 responses *in vivo* during inflammatory conditions and establishes WSX-1 as a critical and non-redundant regulator of the emergent Th1 effector response during malaria infection.

## Author Summary

The cytokine interleukin 27 (IL-27), a member of the IL-12 family, is produced by cells of the innate immune system and has been shown to exert mainly suppressive effects during a wide range of inflammatory conditions, including malaria infection, where it suppresses the development of CD4^+^ T cell-dependent immunopathology. In this study we show that IL-27 suppresses the production of IFN-gamma by CD4^+^ T cells during blood stage malaria infection by preventing the development of terminally differentiated Th1 cells. We investigated the molecular mechanisms by which IL-27 inhibits the formation of terminally differentiated Th1 cells and found that it does so specifically by restricting IL-12 signals. Importantly, we demonstrate that IL-27 mediates its regulatory effects on the Th1 response through IL-10 and Foxp3^+^ regulatory T cell independent mechanisms. Thus, we have identified a new pathway though which IL-27 signalling regulates the size and quality of the Th1 response during malaria infection, which we believe will have relevance to many other pro-inflammatory conditions. Manipulation of the IL-27 pathway may therefore represent an amenable therapeutic approach during chronic inflammatory disorders.

## Introduction

IL-27, a member of the IL-12 super-family, was initially described as a Th1 polarising cytokine due to its ability to increase the sensitivity of CD4^+^ T cells to IL-12 and to promote T-bet expression **[Reviewed 1,2]**. More recently, however, IL-27 has been shown to exert diverse suppressive effects on CD4^+^ T cells during pro-inflammatory conditions [reviewed **1,2**]. IL-27 limits IFN- γ production by CD4^+^ T cells during various infections [**3]**–[7], attenuates the development, but not necessarily maintenance, of Th17 responses by limiting retinoid-related orphan receptor (ROR)c expression [**8]**–[11] and stimulates IL-10 production by multiple effector CD4^+^ T cell populations [**12]**–[14]. All of these effects are mediated via Signal Transducers and Activators of Transcription (STAT) 1 and/or STAT 3 dependent pathways. Finally, IL-27 orchestrates the development of adaptive, IL-10-producing regulatory T cell subsets through induction of c-MAF, Aryl hydrocarbon Receptor (AhR), inducible T-cell co-stimulator (iCOS) and IL-21 pathways [**15]**, [16]. IL-27 is thus a key cytokine that shapes the direction and strength of the T cell response.

Despite reports describing the capacity of IL-27 to limit IFN-γ production by CD4^+^ T cells during inflammation [**3]**–[7], very little work has been performed to understand the molecular basis of this regulatory pathway *in vivo*. IL-27 does not appear to regulate the initial priming or differentiation of Th1 cells during infection [**3]**, [7], unless IL-4 is present, when IL-27 is required to limit Th2 differentiation and enable Th1 responses to develop [**17**]. Thus, IFN-γ production by CD4^+^ T cells is essentially unaltered in IL-27R deficient (WSX-1^−/−^) mice during the early stages of many infections and excessive IFN-γ production, in general, only occurs after day 10 [**3]**, [7] suggesting that WSX-1 regulates established effector CD4^+^ T cells rather than naive or newly primed cells. This temporal control may relate to disparate downstream STAT signalling of the IL-27 receptor in naive and effector CD4^+^ T cells [**18**].

It is possible that WSX-1 signalling could regulate the effector phase of the Th1 response by suppressing the proliferation and expansion of effector Th1 cells and/or by promoting apoptosis of effector Th1 cells, in both cases reducing the magnitude of the Th1 response. Alternatively, WSX-1 could subvert or destabilise the Th1 differentiation programme in maturing Th1 cells, converting Th1 cells into non-Th1 cells [**19**]. Whilst IL-27 has been shown to limit IL-2 production and therefore inhibit Th1 proliferation *in vitro* [**20]**, [21], the role of IL-27 in promoting Th1 cell apoptosis or controlling Th1 cell programming has not been investigated. Moreover the specific pathways through which WSX-1 may modulate these processes in Th1 cells *in vivo* during infection remain poorly described

To define the molecular pathways by which WSX-1 regulates emergent Th1 responses during inflammation, we have utilised the *Plasmodium berghei* (*P. berghei*) NK65 model of murine malaria. We have previously shown that WSX-1 signalling suppresses IFN-γ production by CD4^+^ T cells during this infection and that WSX-1 is essential for preventing CD4^+^ T cell dependent immunopathology [**7**]. We now demonstrate that Th1 priming and the early effector phase of the Th1 response are unaffected by lack of IL-27 signalling during *P. berghei* NK65 infection, but that in WSX-1^−/−^ mice the Th1 response fails to reach a plateau after day 9 of infection leading to the formation of Killer cell Like Receptor Group 1 (KLRG-1)-expressing, terminally differentiated, Th1 cells. Thus, IL-27 signalling constrains the developing Th1 immune response during malaria infection by establishing an upper threshold limit of T-box transcription factor TBX21 (T-bet) expression and suppressing the Th1 molecular programme. Finally we provide mechanistic evidence that IL-27 signalling controls the magnitude and pathogenic activity of the Th1 response by limiting IL-12 dependent signals and that this is independent of IL-10 and Foxp3 regulatory mechanisms. Our data thus provide important new information on how IL-27 regulates CD4^+^ T cell responses during infection.

## Results

### WSX-1 signalling establishes a restrictive threshold for the Th1 response during malaria infection

**Figure 1:**
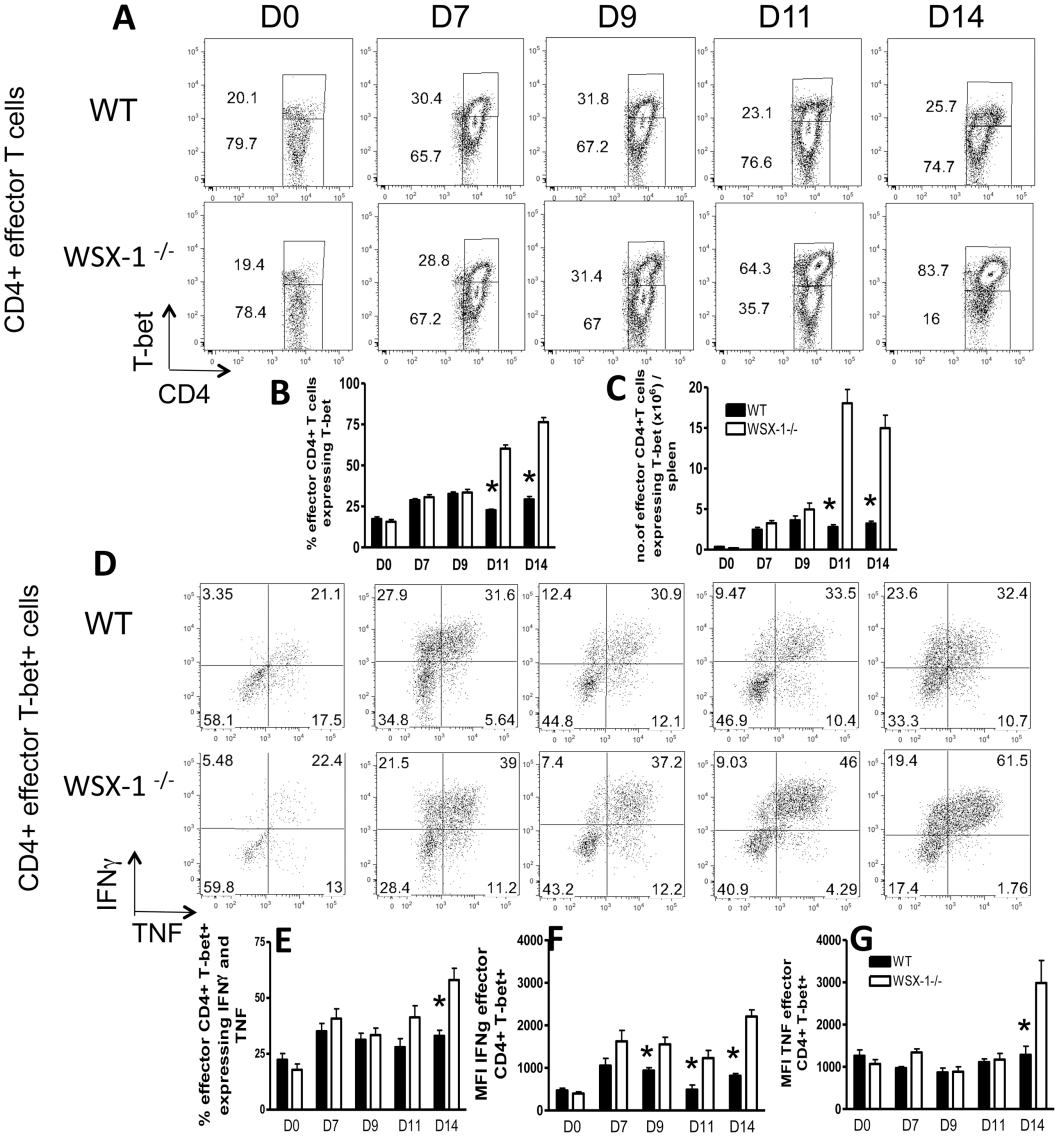
WSX-1 signalling restrains the Th1 response during malaria infection. WT and WSX-1^−/−^ mice were infected i.v. with 10^4^ with *P. berghei* NK65 pRBC. (A) Representative plots showing T-bet expression by splenic CD4^+^ effector (CD44^+^ CD62L^−^) T cells from naïve and infected WT and WSX-1^−/−^ mice. (B, C) The (B) frequencies and (C) total numbers of splenic CD4^+^ effector T-bet^+^ T cells in WT and WSX-1^−/−^ mice. (D) Representative plots showing IFN-γ and TNF expression by splenic Th1 effector CD4^+^ T cells from naïve and infected WT and WSX-1^−/−^ mice following *in vitro* PMA and ionomycin stimulation. (E–F) The Mean fluorescence intensity of (E) TNF and (F) IFN-γ expression by CD4^+^ effector Th1 cells from naïve and infected WT and WSX-1^−/−^ mice. (G) The frequencies of polyfunctional CD4^+^ effector Th1 cells expressing IFN-γ and TNF within the spleen of naïve and infected WT and WSX-1^−/−^ mice. The results are the mean +/− SEM of the group with 3–5 mice per group. The results are representative of 5 independent experiments. * P<0.05 between WT and WSX-1^−/−^ mice.

To investigate whether WSX-1 suppresses IFN-γ production by effector CD4^+^ T cells during malaria infection by down regulating classical Th1 responses, we compared expression of the prototypic Th1-associated transcription factor, T-bet, by splenic effector (CD44^+^CD62L^low^) CD4^+^ T cells in *P. berghei* NK65-infected WT and WSX-1^−/−^ mice. WT mice developed a slow, gradually ascending infection and succumbed with hyperparasitaemia between days 20—25 post-infection (p.i.) (Figure S1). In contrast, parasite levels were significantly lower in infected WSX-1^−/−^ mice from day 7 of infection, but WSX-1^−/−^ mice succumbed to infection on day 13/14 with severe and fatal immunopathology (Figure S1). Frequencies and numbers of splenic effector CD4^+^ T-bet^+^ T cells were equivalent in naïve WT and WSX-1^−/−^ mice, showing that there were no intrinsic differences in T cell polarization in WSX-1^−/−^ mice under homeostatic conditions (Figure 1A–C). Percentages and absolute numbers of splenic effector CD4^+^ T-bet^+^ T cells increased at a similar rate in WT and WSX-1^−/−^ mice until day 9 of infection (Figure 1A–C). The effector CD4^+^T-bet^+^ T cell population plateaued, or even contracted slightly, in WT mice from day 9 of infection, whereas the effector CD4^+^T-bet^+^ T cell population continued to expand in WSX-1^−/−^ mice with both frequencies and numbers of effector CD4^+^T-bet^+^ T cells being significantly higher in WSX-1^−/−^ mice than in WT mice on days 11 and 14 (Figure 1A–C). Similarly, significantly higher frequencies of malaria specific splenic effector CD4^+^ T cells produced IFN-γ in WSX-1^−/−^ mice than in WT mice on day 14 of infection (Figure S2A, B), corresponding with higher plasma levels of IFN-γ [**7**]. Thus, loss of WSX-1 signalling leads to dysregulated T-bet expression and exaggerated Th1 responses specifically after day 9 of infection.

To assess whether aberrant IFN-γ production by CD4^+^ T cells in WSX-1^−/−^ mice during infection [**3]**–[7] was simply due to WSX-1-mediated repression of T-bet expression within the effector population, or was also due to the suppression of T-bet^+^Th1 cell functionality on a cell per cell basis, we examined the capacity of splenic T-bet^+^ effector CD4^+^ T cells from WT and WSX-1^−/−^ mice to produce IFN-γ and TNF following *in vitro* Phorbol 12-Myrisate 13-Acetate (PMA)/ionomycin restimulation. Lack of WSX-1 did not affect the proportion of Th1 cells that were IFN-γ^+^, TNF^+^ or both IFN-γ^+^TNF^+^ on days 0, 7 or 9 of infection (Figure 1D, E and results not shown); however, significantly higher frequencies of T-bet^+^Th1 cells derived from WSX-1^−/−^ mice co-produced IFN-γ and TNF on day 14 of infection (Figure 1D, E) compared with cells from WT mice. Moreover, T-bet^+^ Th1 cells from WSX-1^−/−^ mice produced significantly more IFN-γ on a per cell basis (as measured by mean fluorescence intensity (MFI) of IFN-γ expression) on days 9, 11 and 14, and significantly more TNF on day 14 of infection (Figure 1D, G). Similarly, T-bet^+^ Th1 cells from infected WSX-1^−/−^ (D14 p.i.) mice produced significantly more IFN-γ on a per cell basis when stimulated with malaria antigen compared with cells from WT mice (Figure S2C, D). Thus, from day 9 of infection onwards, WSX-1 not only restricts the magnitude of the Th1 population (by limiting T-bet expression), but also constrains the quality and effector functionality of malaria-specific T-bet^+^ Th1 cells on a cell-per-cell basis.

### WSX-1 signalling represses the development of KLRG-1^+^ Th1 cells during malaria infection

**Figure 2:**
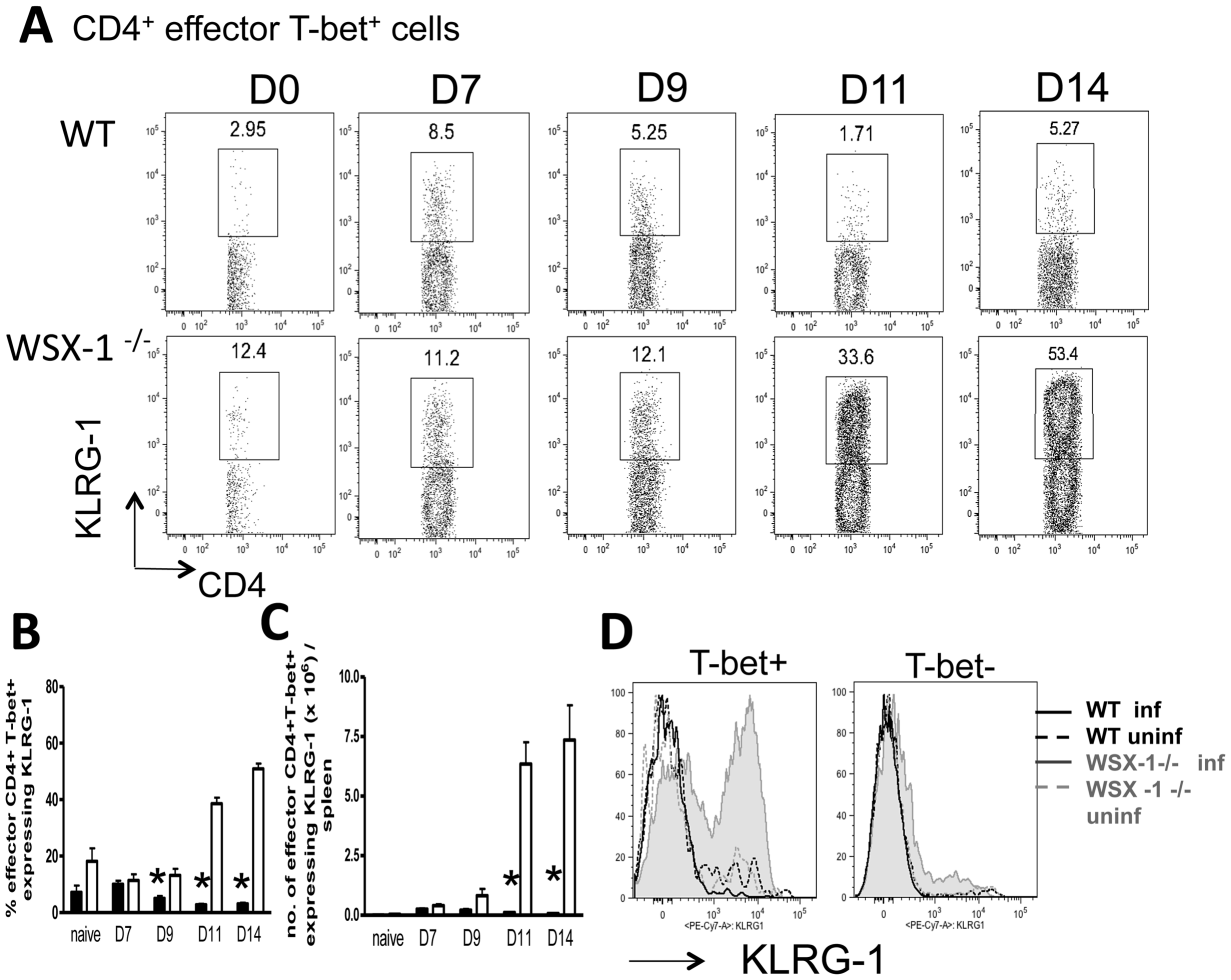
WSX-1 signalling inhibits the development of terminally differentiated KLRG-1^+^ Th1 cells during malaria infection. WT and WSX-1^−/−^ mice were infected i.v. with 10^4^ *P. berghei* NK65 pRBC. (A) Representative plots showing KLRG-1 expression by splenic Th1 effector CD4^+^ T cells from naïve and infected WT and WSX-1^−/−^ mice. (B, C) The (B) frequencies and (C) total numbers of splenic Th1 effector CD4^+^ T cells from naïve and infected WT and WSX-1^−/−^ mice expressing KLRG-1. (D) Representative histograms showing the expression of KLRG-1 by CD4^+^ effector T-bet^+^ and T-bet^−^ T cells derived from naïve (dashed) and D14 infected (solid line) WT (black) and WSX-1^−/−^ (grey) mice. The results are the mean +/− SEM of the group with 3–5 mice per group. The results are representative of 7 independent experiments. * P<0.05 between WT and WSX-1^−/−^ mice.

Our results show that WSX-1 signalling does not restrict the Th1 response during malaria infection by suppressing cellular proliferation or promoting apoptosis (Figures S3, S4). We therefore hypothesised that the temporal dysregulation in the magnitude (and quality) of the Th1 response in malaria-infected WSX-1^−/−^ mice was a direct consequence of the reinforcement of Th1 molecular programming in WSX-1^−/−^ mice, potentiating T-bet expression and terminal differentiation of effector CD4^+^T-bet^+^ T cells. To address this hypothesis, we determined the maturation status of Th1 cells during the course of malaria infection in WT and WSX-1^−/−^ mice by measuring expression of the terminal differentiation marker, KLRG-1. T cell terminal differentiation occurs under strong and continuous polarising signals [**22]**–[24] and, although short lived, terminally differentiated cells are likely to be more stable than incompletely polarised cells [**19]**, [25]. Very few effector CD4^+^ T-bet^+^ T cells expressed KLRG-1 in either WT or WSX-1^−/−^ mice on days 0, 7 and 9 of infection (Figure 2A–C). Similarly, the vast majority of effector CD4^+^ T-bet^+^ cells from WT mice failed to express KLRG-1 on days 11 or 14 infection (Figure 2A–C). In contrast, in WSX-1^−/−^ mice the frequencies, and correspondingly the total numbers, of effector CD4^+^ T-bet^+^ T cells expressing KLRG-1 rapidly increased between day 9 and day 11 of infection, such that more than 50% of all splenic effector CD4^+^ T-bet^+^ cells expressed KLRG-1 on day 14 of infection (Figure 2A–C). Thus, abrogation of WSX-1 signalling led to the maturation and terminal differentiation of a large proportion of the Th1 cell population during malaria infection concomitant with the increase in frequencies and total numbers of splenic Th1 cells after day 9 of infection (Figure 1A–C). Intriguingly, KLRG-1 expression was almost entirely restricted to the effector CD4^+^ T-bet^+^ population and very few T-bet^−^effector CD4^+^ T cells expressed KLRG-1 in either WT or WSX-1^−/−^ mice on day 14 of infection (Figure 2D). KLRG-1 expressing Th1 cells in infected WSX-1^−/−^ mice appeared highly proliferative but were not more potent sources of IFN-γ or TNF than the KLRG-1^−^ Th1 cells on any examined day following PMA/ionomycin stimulation (Figure S5), and produced only slightly more IFN-γ on day 14 of infection following malaria-antigen stimulation (results not shown), suggesting that they may be atypical terminally differentiated cells.

### Ablation of WSX-1 signalling modulates T cell intrinsic expression of stimulatory and inhibitory receptors

**Figure 3:**
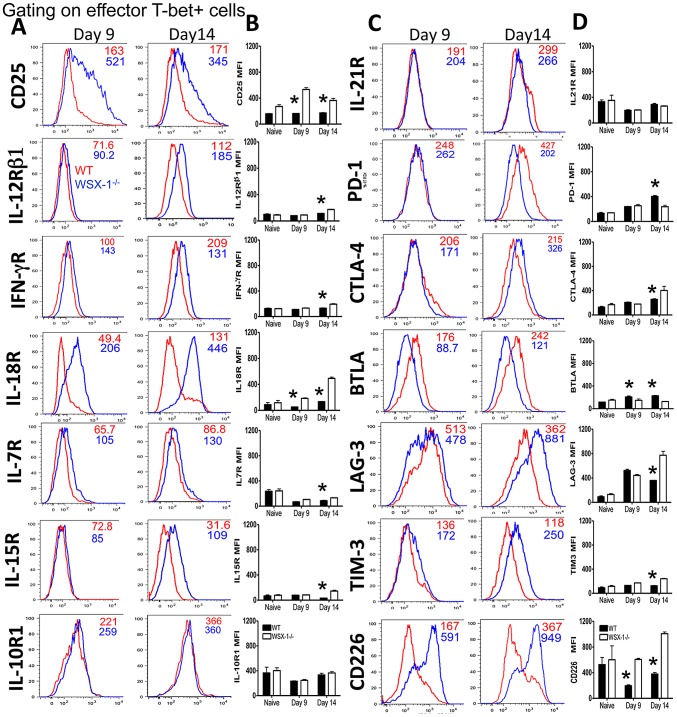
Phenotypic profiling of T-bet^+^ cells in WT and WSX-1^−/−^ mice reveals dysregulated expression of multiple disparate pathways. WT and WSX-1^−/−^ mice were infected i.v. with 10^4^ *P. berghei* NK65 pRBC. (A–D) Expression of cytokine receptors and regulatory receptors by splenic Th1 effector CD4^+^ T cells from naïve and infected WSX-1^−/−^ mice. (A,C) Representative histograms showing expression of each receptor by splenic Th1 effector CD4+ T cells from WT (red lines) and WSX-1^−/−^ mice (blue lines) on days 9 and 14 of infection. (B,D) The mean fluorescence intensity of receptor expression by splenic Th1 effector CD4^+^ T cells from naïve and infected WT and WSX-1^−/−^ mice. The results are the mean +/− SEM of the group with 3–5 mice per group. The results are representative of 4 independent experiments. * P<0.05 between WT and WSX-1^−/−^ mice.

To identify the molecular pathways through which WSX-1 represses Th1 cell terminal differentiation and thereby restricts the magnitude of the Th1 response during infection, we performed a phenotypic analysis of the splenic effector CD4^+^ T-bet^+^ T cells in WT and WSX-1^−/−^ mice immediately prior to (day 9) and following (day 14) dysregulation of the Th1 response in WSX-1^−/−^ mice. We observed no differences in expression (MFI) in any of the examined molecules by effector CD4^+^T-bet^+^ T cells derived from naïve WT and WSX-1^−/−^ mice, demonstrating that there were no intrinsic differences in the regulation of effector CD4^+^ T-bet^+^ T cells in naïve WSX-1^−/−^ mice (Figure 3). Similarly, phenotypes of effector CD4^+^ T-bet^+^ T cells from WT and WSX-1^−/−^ mice were similar on day 9 of infection, with the notable exception of CD25 (IL-2Rα), IL-18R and CD226 which were all expressed at significantly higher levels on cells from WSX-1^−/−^ mice, and B and T lymphocyte attenuator (BTLA), which was expressed at lower levels on cells from WSX-1^−/−^ mice (Figure 3A–D). In contrast, on day 14 of infection, CD25, IL-12Rβ1, IFN-γR, IL-18R, IL-15R, cytotoxic T lymphocyte antigen-4 (CTLA-4), Lymphocyte activation gene-3 (LAG-3), T cell immunoglobulin and musin domain containing protein-3 (Tim-3) and CD226, were all expressed at higher levels by effector CD4^+^ T-bet^+^ T cells derived from WSX-1^−/−^ mice compared to cells from WT mice, whereas Programmed cell death protein 1 (PD-1) and BTLA were both expressed at lower levels by cells from WSX-1^−/−^ mice (Figure 3A–D). CD28, iCOS, 4-1BB and CD27 were expressed at comparable levels on effector CD4^+^ T-bet^+^ T cells from WT and WSX-1^−/−^ mice (results not shown). CD25, IL-12Rβ1, IL-15R, IL-21R, LAG-3, TIM-3 and CD226 were all expressed at higher levels by Th1-KLRG-1^+^ T cells than by Th1-KLRG-1^−^ T cells from WSX-1^−/−^ mice, but the differences in expression were less than the differences observed between Th1 cells from infected WT and WSX-1^−/−^ mice (Figure S6). Thus, dysregulation of the Th1 response in WSX-1^−/−^ mice during malaria infection is associated with temporal and cell-intrinsic changes in multiple stimulatory and inhibitory pathways that could independently or synergistically affect Th1 cell maturation and/or function.

### Th1 cells from infected WSX-1^−/−^ mice are hyperresponsive to IL-12 and IL-2

**Figure 4:**
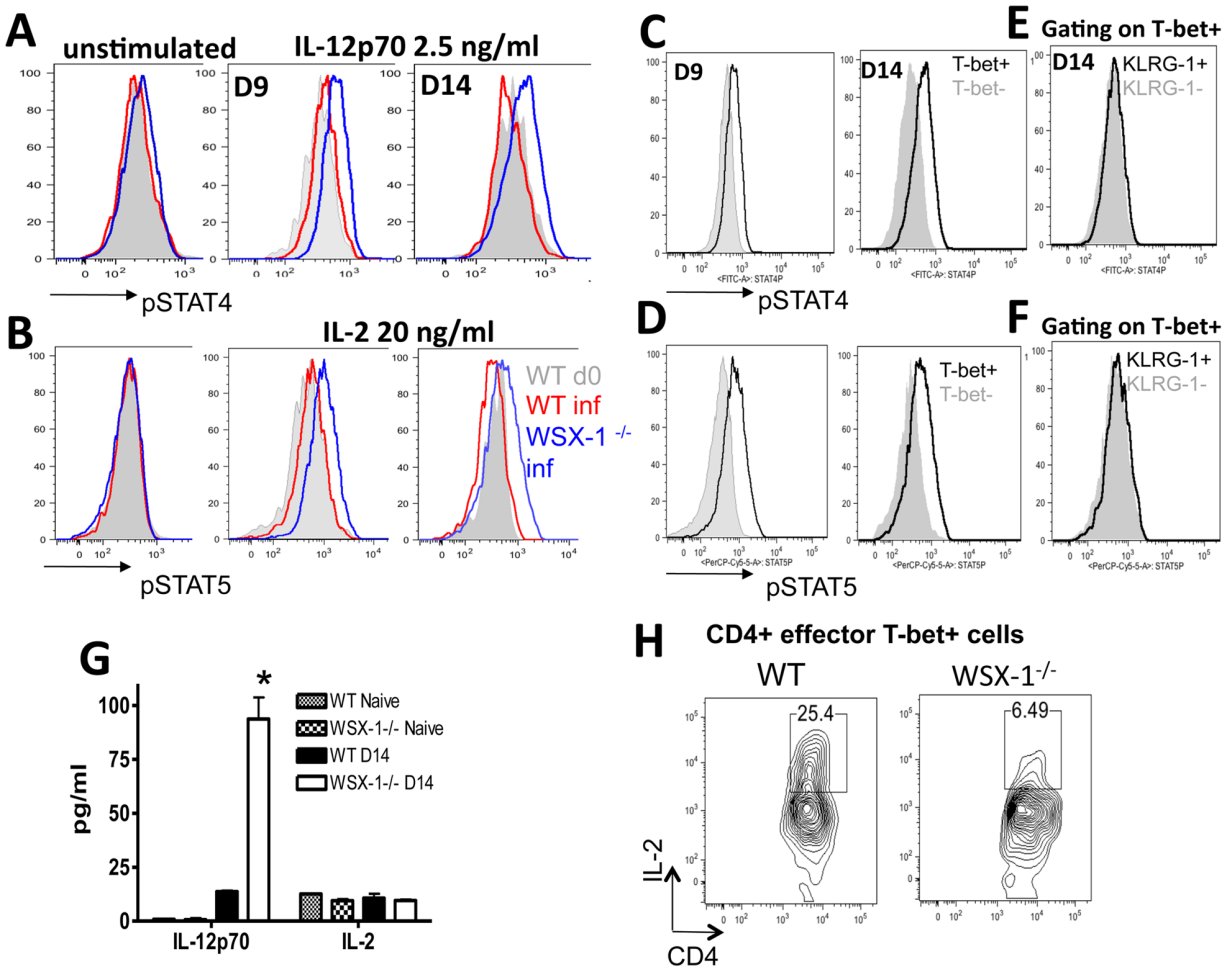
Splenic Th1 cells from infected WSX-1^−/−^ mice are hyper-responsive to IL-12 and IL-2. (A–F) Splenocytes derived from naïve and *P. berghei* NK65 infected (day 9 and 14) WT and WSX-1^−/−^ mice were treated with (A, C, E) rIL-12p70 or (B, D, F) rIL-2 for 10 minutes and the responsiveness of effector CD4^+^ T cell populations was determined by the levels of pSTAT4 and pSTAT5 expression respectively. (A, B) Representative histograms demonstrating the level of (A) pSTAT4 and (B) pSTAT5 expression in Th1 effector CD4^+^ T cells from WT and WSX-1^−/−^ mice following rIL-12p70 and rIL-2 stimulation respectively. (C, D) Representative histograms demonstrating the level of (C) pSTAT4 and (D) pSTAT5 in T-bet^+^ and T-bet^−^ effector CD4^+^ T cells from infected WSX-1^−/−^ mice following rIL-12p70 and rIL-2 stimulation respectively. (E, F) Representative histograms demonstrating the level of (E) pSTAT4 and (F) pSTAT5 in T-bet^+^KLRG-1^+^ and T-bet^+^KLRG-1^−^ effector CD4^+^ T cells from infected WSX-1^−/−^ mice following rIL-12p70 and rIL-2 stimulation respectively. (G) The plasma levels of IL-12p70 and IL-2 in naïve and infected (D14) WT and WSX-1^−/−^ mice, as measured by cytokine bead array. (H) Splenic CD4^+^ T cells purified from WT and WSX-1^−/−^ mice on day 14 of infection were restimulated *in vitro* with anti-CD3 and the frequencies of CD4^+^ effector Th1 cells expressing Il-2 was determined after 4 days by intracellular staining. (G) The results are the mean +/− SEM of the group with 3–5 mice per group. * P<0.05 between infected WT and WSX-1^−/−^ mice. (A–F) The results are representative of 4 independent experiments.

CD25 and IL-12Rβ1 were significantly upregulated on splenic Th1 cells from WSX-1^−/−^ mice compared with cells from WT mice on Day 14. IL-12 is a well-characterised Th1-promoting signal and IL-2 is a T cell growth factor, both of which have also been shown to promote the development of terminally differentiated CD8^+^ T cells [**22]**–[24]. Thus, we examined the functional relevance of upregulated IL-12R and IL-2R expression on effector CD4^+^ T-bet^+^ T cells in promoting Th1 terminal differentiation and hyperactivity in WSX-1^−/−^ mice. We first assessed whether effector CD4^+^ T-bet^+^ T cells from WSX-1^−/−^ mice were hyper-responsive to IL-2 and IL-12. Unstimulated (*ex vivo*) Th1 cells from WT and WSX-1^−/−^ mice on day 14 of infection displayed equivalent levels of pSTAT4 and pSTAT5 expression (Figure 4A, B). In contrast, effector CD4^+^ T-bet^+^ T cells from WSX-1^−/−^ mice on days 9 and 14 of infection were hyperresponsive to both rIL-12p70 and rIL-2, and significantly upregulated pSTAT4 and pSTAT5 respectively following *in vitro* stimulation (Figure 4A, B). In comparison, effector CD4^+^ T-bet^+^ T cells from infected WT mice did not significantly upregulate pSTAT4 or pSTAT5 following rIL-12 or rIL-2 activation (Figure 4A, B). T-bet^+^ effector CD4^+^ T cells from WSX-1^−/−^ mice preferentially responded to both IL-12 and IL-2 compared with T-bet^−^ effector CD4^+^ T cells from WSX-1^−/−^ mice, demonstrating that IL-2 and IL-12 hyperresponsiveness was restricted to the Th1 (T-bet^+^) lineage of cells (Fig. 4 C, D), but there was no differences in the responsiveness of Th1-KLRG-1^+^ and Th1-KLRG-1^−^ cells from WSX-1^−/−^ mice to either IL-2 or IL-12p70 (Figure 4E, F). Nevertheless, IL-12Rβ1^−^ and IL-2R^−^ (CD25^−^) Th1 cells from WSX-1^−/−^ mice (day 14 of infection) responded poorly to IL-12 and IL-2 activation, confirming the functional relevance of increased cytokine receptor expression by Th1 cells in WSX-1^−/−^ mice (results not shown). In line with these data, plasma IL-12p70 concentrations were significantly higher in WSX-1^−/−^ mice than in WT mice on day 14 of infection (Figure 4G). Multiple innate MHC-II^+^ cell populations produced higher amounts of IL-12 in infected WSX-1^−/−^ mice (day 13 of infection) compared with corresponding cells from WT mice, indicating that WSX-1 signalling, directly or indirectly, broadly suppresses the innate compartment during infection; however, CD8^+^ DCs appear to be the most potent source of IL-12 in infected WSX-1^−/−^ mice (Figure S7A). Increased numbers of macrophages and dendritic cells were also observed in the spleens of infected WSX-1^−/−^ mice (D13 p.i) than in infected WT mice (Figure S7B, C). Surprisingly, however, plasma IL-2 concentrations did not differ between infected (D14) WT and WSX-1^−/−^ mice (Figure 4G) and CD4^+^ T cells from infected (D14 p.i) WSX-1^−/−^ mice produced significantly less IL-2 than CD4^+^ cells derived from infected WT mice (Figure 4H).

### Neutralisation of IL-12p40, but not IL-2, attenuates the Th1 response in WSX-1^−/−^ mice during malaria infection

**Figure 5:**
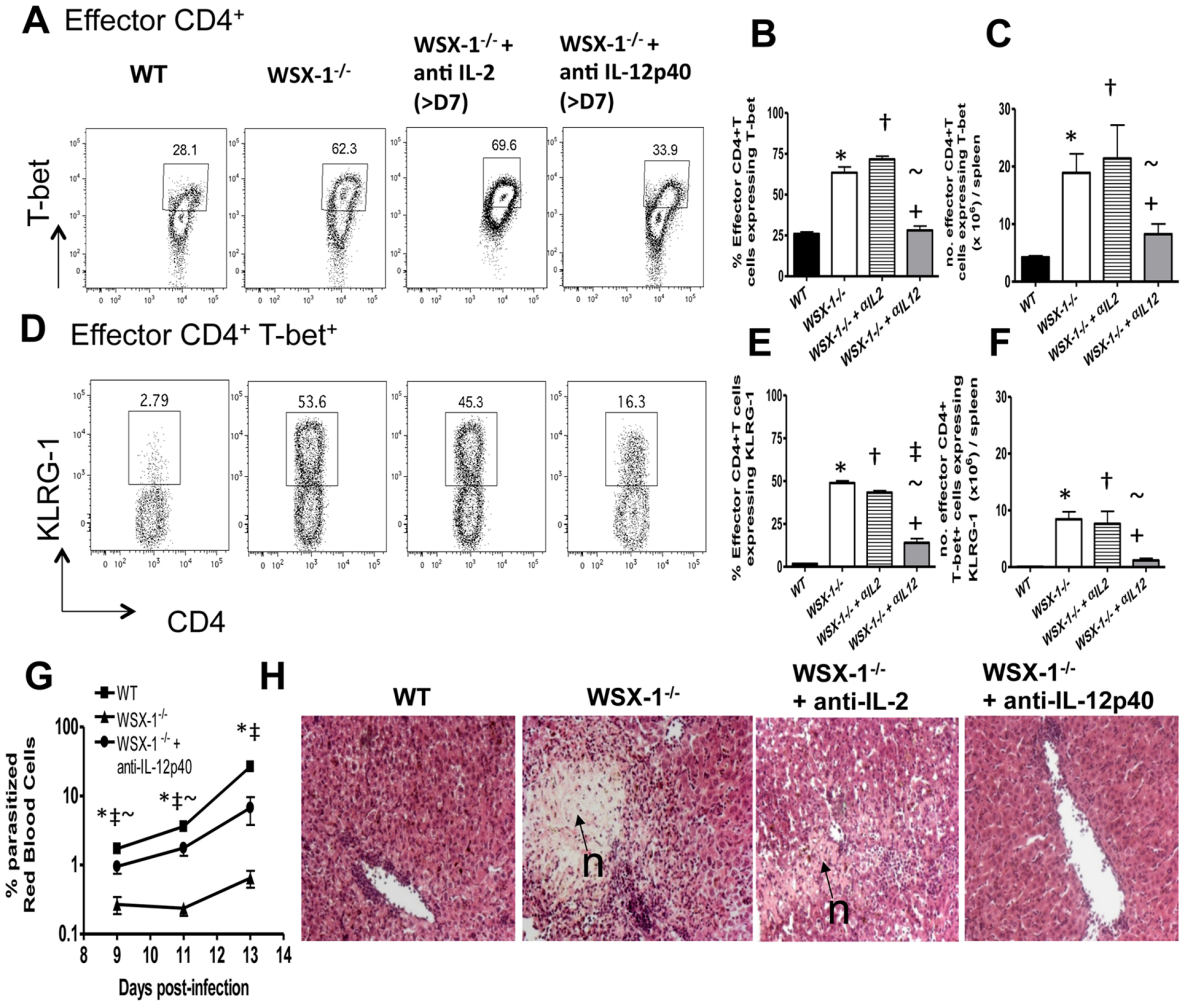
Neutralisation of IL-12p40, but not IL-2, attenuates the Th1 response in WSX-1^−/−^ mice during malaria infection. WT and WSX-1^−/−^ mice were infected i.v. with 10^4^ *P. berghei* NK65 pRBC. (A–F) Groups of WSX-1^−/−^ mice were injected with 250 µg of anti-IL-12 or anti-IL-2 on days 7, 9, 11 and 13 of infection, or isotype Abs in control groups. (A, D) Representative plots from day 14 of infection showing (A) T-bet expression by splenic CD4^+^ effector T cells and (D) KLRG-1 expression by Th1 effector CD4^+^ T cells from WT, WSX-1^−/−^ and treated WSX-1^−/−^ mice. The frequencies (B, E) and total numbers (C, F) of splenic effector and effector Th1 CD4^+^ T cells expressing (B, C) T-bet and (E, F) KLRG-1, respectively. (G) The peripheral parasite burdens in WT, WSX-1^−/−^ and ani-IL-12p40 treated WSX-1^−/−^ mice were assessed on thin smears by microscopy. (H) The level of hepatic pathology in WT, WSX-1^−/−^ mice and WSX-1^−/−^ mice treated with ani-IL-2 or anti-IL-12p40 mAbs was examined on day 13 of infection by histological examination of H & E stained tissue sections (20x magnification). n = areas of necrosis. The results are the mean +/− SEM of the group with 3–5 mice per group. The results are representative of 4 independent experiments. * P<0.05 between WT and WSX-1^−/−^ mice; † P<0.05 between WT and ani-IL-2mAb treated WSX-1^−/−^ mice; ‡ P<0.05 between WT and anti-IL-12p40 mAb treated WSX-1^−/−^ mice; ∼P<0.05 between ani-IL-2mAb treated WSX-1^−/−^ mice and anti-IL-12p40 mAb treated WSX-1^−/−^ mice.

We next determined whether IL-12 and/or IL-2 signals led to over-expansion and terminal differentiation of Th1 cells in WSX-1^−/−^ mice during malaria infection. Administration of anti-IL-2 mAb to WSX-1^−/−^ mice from day 7 of infection (when Th1 responses are similar in WT and WSX-1^−/−^ mice) failed to restrict the Th1 response; frequencies and total numbers of effector CD4^+^ T-bet^+^ T cells (Figure 5A–C), as well as frequencies and numbers of KLRG-1^+^ effector CD4^+^T-bet^+^ T cells (Figure 5D–F), were similar in WSX-1^−/−^ mice treated with anti-IL-2 and control (untreated) WSX-1^−/−^ mice. In contrast, anti-IL-12p40 treatment from day 7 of infection (beginning immediately prior to increase in IL-12 production in WSX-1^−/−^ mice) significantly reduced the frequencies and numbers of effector CD4^+^ T-bet^+^ T cells (to WT levels) and repressed the development of KLRG-1^+^ terminally differentiated cells (Figure 5A–F). Anti-IL-12p40 treatment did not affect the frequencies or numbers of effector CD4^+^ T-bet^+^ T cells in WT mice (results not shown). Clodronate liposome administration from day 7 of infection, which depleted both macrophage and dendritic cell populations, significantly suppressed IL-12p70 production and consequently also reduced Th1 cell terminal differentiation (Figure S**7**D–I). Crucially, whilst anti-IL-2 treatment did not modulate parasite burdens (results not shown), and consequently did not prevent development of fatal immunopathology, anti-IL-12p40 treatment negatively affected parasite control but significantly reduced the level of tissue-immunopathology (Figure 5G, H). Thus, WSX-1 signalling establishes a restrictive threshold for the emergent Th1 response during infection, preventing pathogenic terminal differentiation of Th1 cells, by repressing IL-12p40-dependent signals.

### IL-10 does not restrict T-bet expression by effector CD4^+^ T cells or prevent Th1 cell terminal differentiation during malaria infection

**Figure 6:**
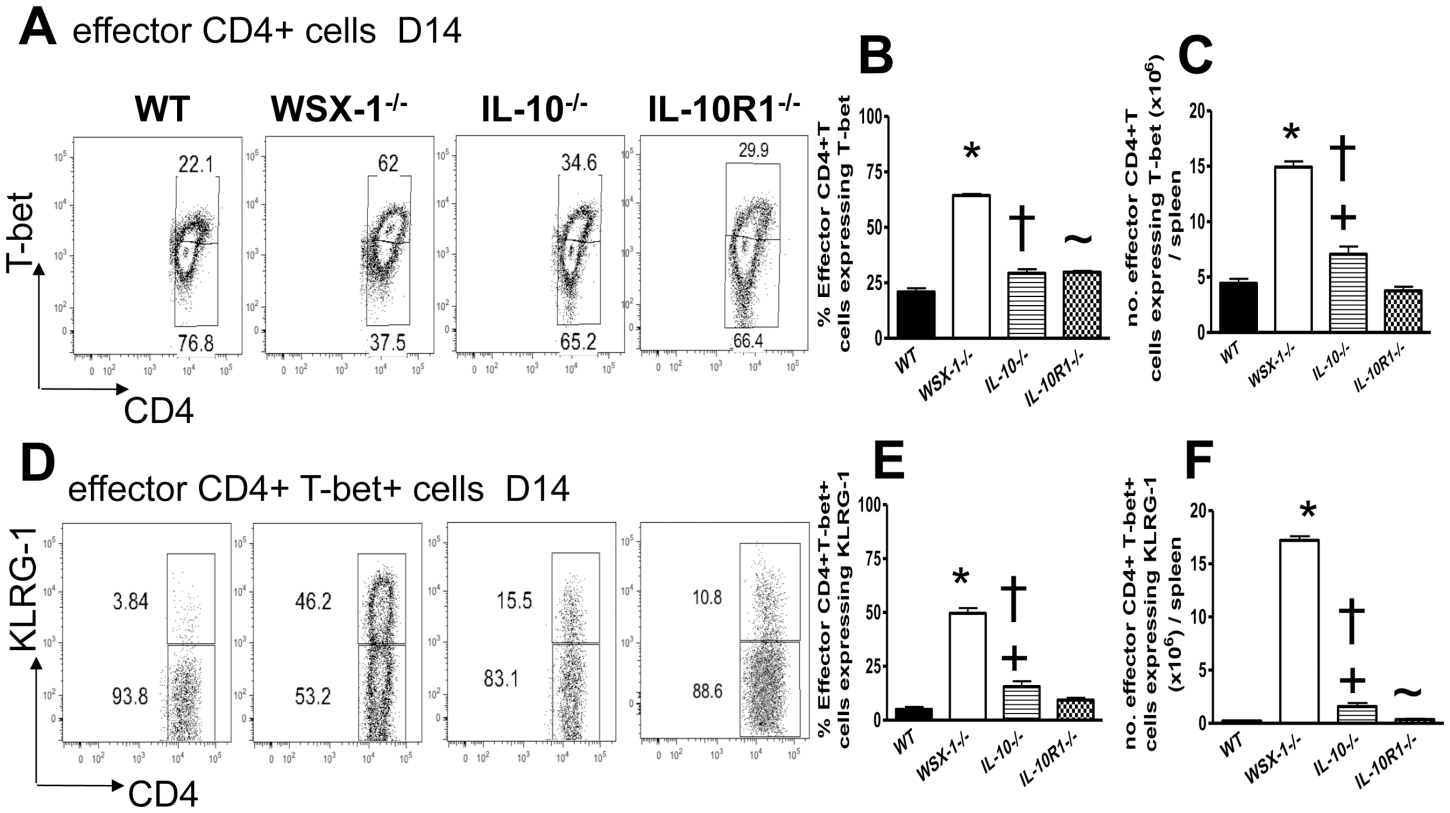
IL-10 does not control the magnitude or terminal differentiation of the Th1 response during malaria infection. WT, WSX-1^−/−^, IL-10^−/−^ and IL-10R1^−/−^ mice were infected i.v. with 10^4^ *P. berghei* NK65 pRBC. (A) Representative plots showing T-bet expression by splenic CD4^+^ effector T cells from each strain of mice on day 14 of infection. (B–C) The (B) frequencies and (C) total numbers of splenic effector CD4^+^ T cells from each strain of mice expressing T-bet on day 14 of infection. (D) Representative plots showing KLRG-1 expression by splenic Th1 effector CD4^+^ T cells from each strain of mice on day 14 of infection. (E, F) The (E) frequencies and (F) total numbers of effector T-bet^+^ CD4^+^ T cells from each strain of mice expressing KLRG-1 on day 14 of infection. The results are the mean +/− SEM of the group with 3–5 mice per group. The results are representative of 3 independent experiments. * P<0.05 between WT and WSX-1^−/−^ mice; † P<0.05 between WSX-1^−/−^ and IL-10^−/−^ mice; ∼P<0.05 between WSX-1^−/−^ and IL-10R1^−/−^ mice; +P<0.05 between WT and IL-10^−/−^ mice.

IL-27 promotes IL-10 production by various populations of T cells during inflammatory conditions [**12]**–[16]. As we, and others, have shown an important role for IL-10 in limiting immunopathology during malaria infection [**7]**, [14], [26], [27], we determined whether elevated T-bet expression by effector CD4^+^ T cells and increased Th1 cell terminal differentiation in WSX-1^−/−^ mice during infection was due to lack of IL-10. Intriguingly, ablation of IL-10 production and IL-10R1 expression did not lead to a marked increase in the frequencies or total numbers of effector CD4^+^T-bet^+^ T cells during infection (Figure 6A–C). Consistent with this, lack of IL-10 and IL-10R1 led to only a marginal increase in frequencies and total numbers of KLRG-1^+^ effector CD4^+^T-bet^+^ T cells and their numbers were significantly lower in IL-10^−/−^ and IL-10R1^−/−^ mice than in WSX-1^−/−^ mice (Figure 6D–F). Thus, these data strongly indicate that WSX-1 signalling controls pathogenic Th1 responses during malaria infection through IL-10 independent mechanisms.

### The Foxp3^+^ Treg response is largely unaltered in WSX-1^−/−^ mice during malaria infection

**Figure 7:**
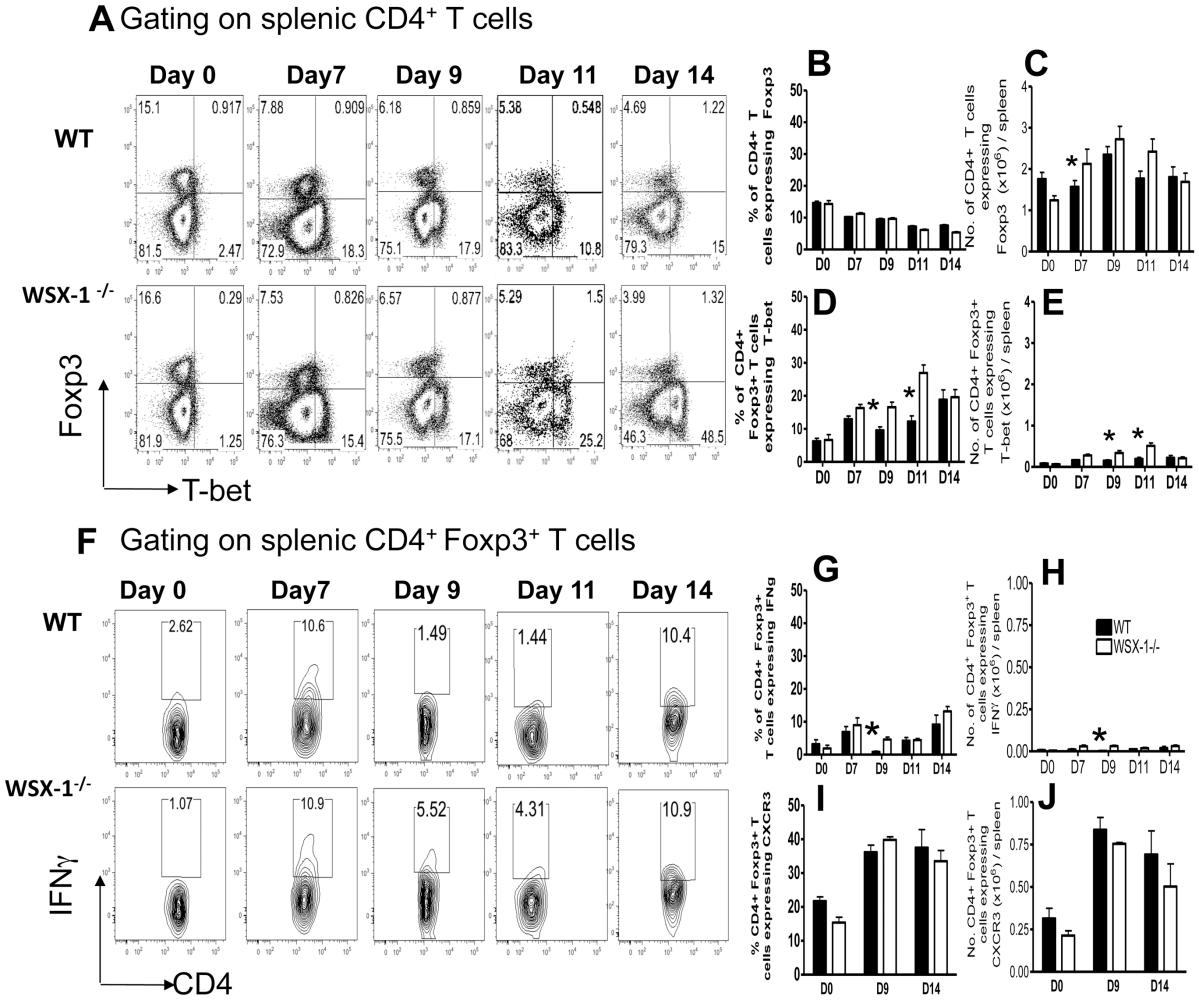
The Foxp3^+^ Treg response is unaltered in WSX-1^−/−^ mice during malaria infection. WT and WSX-1^−/−^ mice were infected with *P. berghei* NK65. (A) Representative plots showing the expression of Foxp3 vs Tbet by splenic CD4^+^ T cells from naive and infected WT and WSX-1^−/−^ mice. (B–E) The (B, D) frequencies and (C, E) total numbers of splenic CD4^+^ T cells from naive and infected WT and WSX-1^−/−^ mice expressing FoxP3 and splenic CD4^+^FoxP3^+^ T cells from naive and infected WT and WSX-1^−/−^ mice expressing T-bet, respectively. (F) Representative plots showing the expression of Foxp3 vs IFN-γ by splenic CD4^+^ T cells from naive and infected WT and WSX-1^−/−^ mice following *in vitro* stimulation with PMA and ionomycin. (G–H) The (G) frequencies and (H) total numbers of splenic CD4^+^FoxP3^+^ T cells from naive and infected WT and WSX-1^−/−^ mice expressing IFN-γ. (I–J) The (I) frequencies and (J) total numbers of splenic CD4^+^FoxP3^+^ T cells from naive and infected (D14) WT and WSX-1^−/−^ mice expressing CXCR3. The results are the mean +/− SEM of the group with 3–5 mice per group. The results are representative of 3 independent experiments. * P<0.05 between WT and WSX-1^−/−^ mice.

IL-27 can suppress the maintenance and functionality of natural Foxp3^+^ regulatory T cells (Foxp3^+^ Treg) [**28]**–[31]. However, it has also been suggested that Foxp3^+^ Treg numbers can collapse during highly pro-inflammatory events, due to conversion into Th1 cells and apoptosis, initiating a pro-inflammatory feedback loop leading to development of immune mediated pathology [**32**]. Although the role of Foxp3^+^ Treg during malaria is far from clear [**26]**, [33]–[35], Foxp3^+^ Treg can, in other models, regulate Th1 cell homeostasis [**36**]. Thus, as the final part of this study, we determined whether Foxp3^+^ Treg numbers and/or phenotype were modulated in WSX-1^−/−^ mice during malaria infection. The frequencies and absolute numbers of splenic CD4^+^ Foxp3^+^ T cells were largely comparable in WT and WSX-1^−/−^ mice at all time points (Figure 7A–C). Interestingly, the proportion of CD4^+^Foxp3^+^ T cells co-expressing T-bet increased in both WT and WSX-1^−/−^ mice during the course of malaria infection (Figure 7A, D), and significantly higher frequencies and numbers of CD4^+^Foxp3^+^T-bet^+^ T cells were observed in WSX-1^−/−^ mice compared with WT mice on days 9 and 11 of infection, although cell numbers were low (Figure 7D, E). Very few splenic CD4^+^Foxp3^+^ T cells expressed IFN-γ in naïve or malaria-infected WT or WSX-1^−/−^ mice and there was only a transient difference in the frequencies and numbers of CD4^+^Foxp3^+^ IFN-γ^+^ cells in WT and WSX-1^−/−^ mice on day 9 of infection (Figure 7F–H). Moreover, there were no major differences in the frequencies or numbers of CD4^+^Foxp3^+^ T cells expressing CXCR3 (Th1-adapted Treg [**37**] in either the spleen or livers of naive or infected WT and WSX-1^−/−^ mice (Figure 7I, J and results not shown). These data suggest that a small proportion of Foxp3^+^ Treg either polarise to a specialised Th1-regulatory phenotype [**37**], or convert into non-regulatory effector cells [**32**] during malaria infection, and that WSX-1 may play a very minor and transient role in regulating this adaptation. Crucially, however, depletion of Foxp3^+^ regulatory cells throughout the course of malaria infection, using DEREG mice, did not significantly increase the level of Th1 cell differentiation or lead to development of KLRG-1^+^ Th1 cells (Figure S8). Thus, Foxp3^+^ T cells do not regulate the magnitude or terminal differentiation of the Th1 population during malaria infection.

## Discussion

In this study we have defined the molecular mechanisms by which IL-27 restricts Th1 immune responses during infection. Whilst it is well established that WSX-1 signalling limits IFN-γ production by T cells during infection and inflammation [**1]**, [2], our study is the first to identify that it does so specifically by preventing the generation of terminally differentiated KLRG-1^+^ Th1 cells. We have demonstrated that although WSX-1 signalling modulates the expression of multiple stimulatory and inhibitory receptors on Th1 cells during infection, including PD-1 and BTLA, individual neutralisation of IL-12p40 from day 7 of infection was sufficient to prevent aberrant T–bet expression, abrogate the development of terminally differentiated KLRG-1^+^ Th1 cells and attenuate T-cell dependent immunopathology in malaria infected WSX-1^−/−^ mice. Thus, the dominant immunoregulatory role of IL-27 – signalling via WSX-1 - in preventing hyperactive Th1 responses *in vivo* during malaria infection appears to be the downregulation of IL-12-dependent pathways.

We have shown that Th1 cells derived from malaria-infected WSX-1^−/−^ mice are hyper responsive to IL-12p70 and that that IL-12p70 protein levels are significantly higher in WSX-1^−/−^ mice than in WT mice at the later stages of infection, when the Th1 cell responses start to diverge. It has previously been shown that macrophages and dendritic cells derived from WSX-1^−/−^ mice are hyper-responsive to TLR signalling and produce more IL-12p40 [**5]**, [38] than WT cells and that IL-27 reduces IL-12p40 production by macrophages *in vitro* [**5**]. Therefore, it is perhaps unsurprising that we found that macrophages and dendritic cells, in particular the CD8^+^ DC subset that is the dominant source of IL-12 in various other infections [**39**], expressed higher levels of IL-12 in malaria infected WSX-1−/− mice than in infected WT mice. However, T cell intrinsic WSX-1 expression has also been shown to be required to limit T cell proliferation and IFN-γ production *in vivo* during infection [**3**]. Consequently, it is currently unclear whether the dysregulated IL-12 pathway in WSX-1^−/−^ mice during infection, and the corresponding development of KLRG-1^+^ Th1 cells, is due to intrinsic loss of WSX-1 mediated regulation within the innate system, specifically by macrophages and dendritic cells, or whether it is a consequence of abrogated WSX-1 expression on CD4^+^ T cells, which subsequently leads to amplification of the innate immune response, initiating a positive inflammatory feedback loop. We are currently examining the relative importance of CD4^+^ T cell intrinsic and extrinsic WSX-1 signalling in limiting the IL-12 pathway, and hence Th1 cell differentiation, *in vivo* during infection.

Th1 cell proliferation and apoptosis were relatively unaltered in WSX-1^−/−^ mice during the course of malaria infection, suggesting that Th1 hyperactivity in WSX-1^−/−^ mice during malaria infection was not due to differences in cellular expansion or survival. It has previously been shown that malaria infection promotes a biphasic effector T cell response in WT mice, with Th1 responses established early in infection being replaced by Th2-dominant responses later in infection [**40**]. As IL-4 mRNA levels are significantly lower in CD4^+^ T cells from WSX-1^−/−^ mice than from WT mice on day 13 of infection (**7)** our results suggest that the transition from Th1 to Th2 based immunity does not occur in WSX-1^−/−^ mice during malaria infection. Thus, our results indicate that WSX-1 signalling limits Th1 cell terminal differentiation and establishes an upper threshold of T–bet expression within the effector CD4^+^ T cell population by inducing instability within the Th1 molecular programme, causing incompletely polarised Th1 cells – which exhibit significantly higher functional flexibility than repeatedly restimulated Th1 cells [**25**] - to lose T–bet expression and convert into non-Th1 cell populations, such as Th2 cells. Whilst Th1 cells are believed to be more stable than Th17 and iTreg cells [**19**], the signals that reciprocally enforce and oppose the fidelity of the Th1 molecular programme during infection or inflammation *in vivo* are poorly defined [**19**]. Our data indicate that IL-27 may be one such cytokine that orchestrates Th1 cell conversion *in vivo* to reduce immune mediated pathology during infection.

The molecular cues that govern the terminal differentiation of effector CD4^+^ T cells are less well characterised than those that control development of effector CD8^+^ T cells but our data suggest that there are some similarities – and some important differences – between the two processes. Strong and prolonged IL-12, IL-15 and IL-2 (CD25 dependent) signalling induces the graded expression of T-bet and B lymphocyte-induced maturation protein 1 (Blimp1) in CD8^+^ T cells, promoting the development of short-lived, terminally differentiated effector (KLRG-1^+^, CD127^lo^) CD8^+^ T cells at the expense of long-lived memory populations, [**22]**–[24], [41]–[44]. In contrast, our data suggest that IL-12, but not IL-2, is the key cytokine driving Th1 cell terminal differentiation during infection, presumably through STAT4 positive enforcement of T-bet expression. Whilst anti-IL-12p40 treatment also potentially abrogated IL-23 activity we do not believe IL-23 plays a major role in promoting Th1 cell terminal differentiation in malaria-infected WSX-1^−/−^ mice. IL-23 is not overproduced in WSX-1^−/−^ mice during malaria infection and Th17 responses are not amplified in malaria-infected WSX-1^−/−^ mice [**7**], indicating that IL-23 does not exert strong activity in infected WSX-1^−/−^ mice. It is also interesting that loss of IL-27 immunoregulation specifically leads to Th1 cell terminal differentiation during malaria infection and very few non-Th1 cells express KLRG-1. This suggests that there is as specific imbalance in signals that promote Th1 cell terminal differentiation in WSX-1^−/−^ mice during malaria infection and that disparate cues, which are unaffected in infected WSX-1^−/−^ mice, orchestrate terminal differentiation of other CD4^+^ T cell subsets. Indeed, IL-4 is expressed at lower levels in malaria infected WSX-1^−/−^ mice than in WT mice [**7**]. In addition, it is also possible that during malaria infection direct IL-27R signalling specifically inhibits Th1 molecular programming.

As IL-27 can induce IL-10 production by effector CD4^+^ T cell populations [**7]**, [12]–[16], including during malaria infection [**7]**, [14], we initially hypothesized that the hyperactive Th1 phenotype observed in WSX-1^−/−^ mice would be recapitulated in IL-10^−/−^ or IL-10R1^−/−^ mice. Indeed, we and others have shown that IL-10 is required to limit morbidity and mortality during malaria infection [**7]**, [14], [26], [27]. Surprisingly, however, the Th1 response was quantitatively and qualitatively similar in IL-10R1^−/−^ mice and WT mice during malaria infection. These data strongly suggest that WSX-1 does not regulate Th1 responses *in vivo* during infection specifically through IL-10-dependent mechanisms. Consistent with this, IL-27 has previously been shown to mediate IL-10-independent mechanisms [**13**]. Thus, under physiological conditions, IL-27 and IL-10 appear to have discrete immunoregulatory functions *in vivo* during malaria infection.

We have also shown that the Foxp3^+^ regulatory T cell population is largely unaltered in WSX-1^−/−^ mice during malaria infection; the frequency, absolute number and phenotype (T-bet, CXCR3 and IFN-γ) of Foxp3^+^ Tregs were essentially the same in infected WT and WSX-1^−/−^ mice. Thus, although we cannot be entirely sure that the Foxp3 Tregs maintain their regulatory function during malaria infection in WSX-1^−/−^ mice, there is no evidence that WSX-1 regulates the collapse of the Foxp3^+^ T cell population during malaria infection. Moreover, it does not appear that WSX-1 controls the functional adaptation of Foxp3^+^ Tregs to become Th1-Foxp3^+^ Treg (CXCR3^+^Foxp3^+^) during malaria infection, as is observed during *T. gondii* infection [**45**]. Irrespective of the role of IL-27 in modifying the nature of the Foxp3^+^ regulatory cell compartment, we have shown that depletion of Foxp3^+^ regulatory T cells throughout the course of malaria infection does not lead to the expansion or terminal differentiation of Th1 cells. Thus, combined, our results strongly indicate that IL-27 controls Th1 responses during malaria infection through Foxp3^+^ regulatory T cell independent mechanisms.

In summary, our study has significantly expanded our understanding of how IL-27/WSX-1 signalling regulates Th1 responses *in vivo* during infection. We have shown that WSX-1 signalling regulates the molecular programming of Th1 cells, inhibiting the formation of terminally differentiated KLRG-1^+^ Th1 cells, and thereby establishes an upper threshold limit of T-bet expression within the CD4^+^ effector T cell population. Importantly, IL-27 mediates its effects independently of IL-10 and Foxp3^+^ Tregs. Thus, our data highlight a critical and non-redundant role for IL-27/WSX-1 signalling in regulating the size and quality of the Th1 response during infection. Manipulation of the IL-27 pathway may therefore represent a therapeutic approach to limit T cell dependent immunopathology and/or enhance pathogen control during chronic inflammatory disorders.

## Materials and Methods

### Ethics statement

All animal work was approved following local ethical review by LSHTM and University of Manchester Animal Procedures and Ethics Committees and was performed in strict accordance with the U. K Home Office Animals (Scientific Procedures) Act 1986 (approved H.O Project Licenses 70/6995 and 70/7293).

### Mice and parasites

C57BL/6 mice were purchased from Charles River, UK. Breeding pairs of C57BL/6 IL-27R knockout (WSX-1^−/−^) mice [**46**] were provided by Amgen Inc (Thousand Oaks, USA). C57BL/6 IL-10^−/−^ and C57BL/6 IL-10R1^−/−^ knockout mice were kindly provided by Professor Werner Muller (University of Manchester). DEREG mice, which express DTR receptor and GFP under control of the FoxP3 promoter [**47**], were kindly provided by Dr Mark Travis (University of Manchester). All mice were maintained at the London School of Hygiene and Tropical Medicine and the University of Manchester. All transgenic mice were fully backcrossed to C57BL/6 background. Sex-matched 6 to 10 weeks old mice were used in separate experiments and maintained in individually ventilated cages.

Cryopreserved *P. berghei* NK65 parasites were thawed and passaged once through C57BL/6 mice before being used to infect experimental animals. Mice were infected intravenously with 10^4^ parasitized red blood cells (pRBC). In some experiments, WSX-1^−/−^ mice were injected intraperitoneally with 250 µg anti-IL-12p40 (clone C17.8), 250 µg anti-IL-2 (JES6-5H4) or 300 µl of clodronate liposomes on days 7, 9, 11 and 13 of infection. Purified rat IgG2a was used to verify the specific *in vivo* activity of anti–IL-12p40 and anti-IL-2 Abs. All Abs were obtained from BioXCell (West Lebanon, NH). DEREG mice and non-transgenic littermates were injected with 200 ng DT i.p. from day −1 and every two days p.i. The course of infection was monitored every 2nd day by microscopic examination of peripheral parasitaemia on Giemsa-stained thin blood smears and by assessing weight loss.

### Flow cytometry

Spleens were collected from naïve and malaria-infected mice (days 7, 9, 11 or 13/14) and single-cell suspensions were prepared by homogenization through a 70 µm cell strainer (BD Biosciences). RBCs were lysed (RBC lysing buffer, BD Biosciences), splenocytes washed and resuspended in FACS buffer (HBSS with 2% FCS). Live/dead cell differentiation and absolute cell numbers were calculated by trypan blue exclusion (Sigma-Aldrich) using a haemocytometer.

CD4^+^ T cells were characterised by surface staining with anti-mouse antibodies against CD4 (GK1.5), CD44 (IM7), CD62L (MEL-14), CXCR3 (CXCR3-173), KLRG1 (2F1), IFNγR1 (2E2), IL-12Rβ1 (114), PD-1 (RMP1-30), BTLA (8F4), CTLA-4 (UC10-4B9), CD25 (PC61), IL-18Rα (BG/IL18RA), IL-7Rα (A7R34), IL-15Rα (DNT15Ra), IL-21R (4A9), LAG-3 (C9B7W), TIM-3 (RMT3-23) and CD226 (10E5). For intracellular staining, surface-stained cells were washed in FACS buffer and permeabilized with Foxp3 fixation/permeabilization buffers (eBioscience) for 30 min. The cells were then washed and stained in FACS buffer with anti-mouse antibodies against T-bet (4B10), Foxp3 (FJK-16s) and CTLA-4 (UC10-4B9) for 30 minutes. To assess intracellular IFN-γ and TNF production, 1×10^6^ live cells were incubated in RPMI 1640 medium supplemented with 10% FCS, 200 ng/ml PMA (Sigma) and 1 µg/ml ionomycin (Sigma) in the presence of Brefeldin A (1∶1000) for 5 h at 37°C, 5% CO_2_. For experiments where specific parasite responses were assessed, T cells were depleted in naïve splenocytes from WT or WSX-1^−/−^ mice using anti-TCRβ PE antibodies and anti-PE conjugated MACS beads (Miltenyi Biotec), according to the manufacturer's instructions. TCR-depleted splenocytes were seeded at 250,000/well and pulsed overnight with 15×10^6^ *P. berghei* NK65 pRBC lysate/ml. Control samples included non-pulsed splenocytes. Cultures were then incubated with 125,000 purified naïve or day 13 infection-derived WT or WSX-1^−/−^ CD4^+^ T cells. IFN-γ levels were assessed by intracellular staining after 18 h culture. To detect intracellular IL-2, 1×10^6^ cells were stimulated with 2 µg/ml of anti-CD3 (BD biosciences) for 96 hrs, followed by PMA and inonomycin restimulation in presence of brefeldin A, as described above. The cells were washed, stained for surface markers CD4 and CD44, permeabilized and stained with anti-mouse IFN-γ (XMG1.2), anti-mouse TNF (MP6-XT22) or anti-mouse IL-2 (JES6-5H4). All antibodies were purchased from eBioscience, Biolegend or BD Biosciences. Fluorescence minus one controls were used to validate flow cytometric results (Figure S9). All flow cytometry acquisition was performed using an LSR II (BD Systems, UK). All FACS analysis was performed using Flowjo Software (Treestar Inc, OR, USA).

### Assessment of cell proliferation, survival and apoptosis

For the analysis of cell proliferation *in vivo*, 1.25 mg sterile BrdU (5-bromodeoxyuridine) diluted in PBS was injected intraperitoneally 1 h before mice were killed and organs harvested. Single cell splenocyte suspensions were prepared and surface molecules stained as described above. Intracellular BrdU incorporation was measured by flow cytometry using an anti-BrdU antibody (clone PRB-1, eBioscience) following the manufacturer's instructions. Cells were co-stained for the nuclear antigen Ki67 (clone B56, BD Biosciences). Survival of naïve (CD62L^high^ CD44^low^) and effector Th1 (CD62^low^ CD44^high^ T-bet^+^) CD4^+^ T cells was assessed by intracellular staining of Bcl-2 (clone BCL/10C4, BioLegend). T cell apoptosis was assessed by flow cytometry using Annexin V (BD biosciences) and fixable viability dye (eBioscience), following the eBioscience Annexin V staining protocol.

### Intracellular staining for phosphorylated STAT4 and STAT5

Splenic single-cell suspensions from uninfected, day 9 and day 14 *P.berghei* NK65 infected C57BL/6 and WSX-1^−/−^ mice were obtained as described above. 1×10^6^ cells/sample were rested on ice in Medium for 30 min. Cells were incubated with 20 ng/ml IL-2 (eBioscience) or 2.5 ng/ml IL-12 (R&D Systems) for 10 min at 37°C, 5% CO_2_ and immediately fixed for 15 min on ice by addition of an equal volume of 4% paraformaldehyde. Cells were permeabilized with 90% ice-cold methanol at −20°C o/n and then stained for CD4, CD44, CD62L, T-bet and phosphorylated STAT4 (at residue Y693, clone 38) or phosphorylated STAT5 (at residue Y694, clone 47; both BD Biosciences) in FACS buffer, washed and analysed by flow cytometry.

### Cytokine quantification

Heparinised blood from uninfected and *P. berghei* NK65 infected C57BL/6, IL-10^−/−^,WSX-1^−/−^, anti-IL-12p40 and clodronate liposomes treated WSX-1^−/−^ mice was collected and spun at 5000 g for 6 minutes. Plasma was immediately stored at −80°C until further use. Plasma IL-12p70, IL-2 and IFN-γ were measured by Cytometric bead array (CBA) assay (BD Biosciences), following the manufacturer's instructions.

### Real-time PCR

Splenic MHC-II^+^ splenocytes from *P. berghei* NK65 infected WT and WSX-1^−/−^ mice (day 13 p.i.) were presorted by positive magnetic selection using anti-MHC-II PE antibody and anti-PE MACS beads (Miltenyi). Cells were stained with a cocktail of antibodies against CD3e (145-2C11), CD8a (53-6.7), CD11b (14.0112.81), CD11c (17-0114-81) and F4-80 (14-4801-81) and different APC populations were sorted using a FACSAria according to the gating strategy described in Figure S7A. mRNA was isolated using the RNeasy isolation kit (Qiagen, Valencia, CA) and DNAse I treated (Ambion/ABI, Austin, TX) prior to cDNA synthesis. IL-12p35mRNA levels were quantified using validated gene expression assays from ABI Biosystems and cDNA expression was standardized using the housekeeping gene â-actin. (Life Technologies Ltd, Paisley UK).

### Histopathology

A section of liver tissue was removed on day 13/14 p.i. from all animal groups and fixed in 10% formalin saline. Fixed tissues were paraffin embedded and sectioned, followed by H&E staining (Independent Histological Services, London, U.K.). Sections were examined under a light microscope using ×20 magnification.

### Statistical analysis

All data were tested for normal distributions using the D'Agostino and Pearson omnibus normality test. In two group comparisons statistical significance was determined using the *t* test or the Mann–Whitney *U* test, depending on distribution of the data. For three or more group comparisons, statistical significance was determined using a one-way ANOVA, with the Tukey post hoc analysis for normally distributed data, or a Kruskal–Wallis test, with Dunn post hoc analysis for nonparametric data. All statistical analyses were performed using GraphPad Prism. Results were considered as significantly different when *p*<0.05.

## Supporting Information

Figure S1 **The course of** ***P. berghei*** **NK65 infection in WT and WSX-1^−/−^ mice.** WT and WSX-1^−/−^ mice were infected i.v. with 10^4^ *P. berghei* NK65 pRBC. The peripheral parasite burdens in WT and WSX-1^−/−^ mice were assessed on thin smears by microscopy. * P<0.05 between WT and WSX-1^−/−^ mice.
(TIF)

Figure S2 **Malaria specific CD4^+^ T cells from infected WSX-1^−/−^ mice produce significantly more IFN-γ than corresponding cells from infected WT mice.** Splenic CD4^+^ T cells were purified from naïve and malaria-infected (day 13 p.i.) WT and WSX-1^−/−^ infected mice and were restimulated *in vitro* with *P. berghei* NK65 pulsed APCs obtained from naïve mice. Representative plots showing IFN-γ expression by CD4^+^ effector T cells (A) or Th1 effector CD4^+^ T cells (C) after overnight restimulation. The frequencies of CD4^+^ effector T cells (B) or Th1 effector CD4^+^ T cells (D) expressing IFN-γ from WT and WSX-1^−/−^ naïve or infection-derived CD4^+^ T cells. The results are the mean +/− SEM of 3 independent wells. * P<0.05 between WT and WSX-1^−/−^ pulsed groups.
(TIF)

Figure S3 **Restriction of splenic Th1 response in WT mice during malaria infection is not due to impaired Th1 cell proliferation.** WT and WSX-1^−/−^ mice were infected i.v. with 10^4^ *P. berghei* NK65 pRBC. 1.25 mg of BrdU was injected i.p. 1 h before animals were culled. (A) Representative plots showing Ki67 expression versus BrdU incorporation by splenic Th1 effector CD4^+^ T cells from naïve and infected WT and WSX-1^−/−^ mice. Numbers within plots represent the frequencies of Ki67^+^ BrdU- cells (top left) and Ki67^+^ BrdU^+^ (bottom right). (B–E) The frequencies (B–C) and total numbers (D–E) of splenic CD4^+^ effector T-bet+ T cells expressing (B, D) Ki67 and (C, E) incorporating BrdU. The results are the mean +/− SEM of the group with 3–5 mice per group. The results are representative of 3 independent experiments. * P<0.05 between WT and WSX-1^−/−^ mice.
(TIF)

Figure S4 **Restriction of splenic Th1 response in WT mice is not due to IL-27R- direct or indirect promotion of Th1 cell apoptosis or altered survival.** WT and WSX-1^−/−^ mice were infected i.v. with 10^4^ *P. berghei* NK65 pRBC. (A) Representative plots showing Annexin V expression by splenic Th1 effector CD4^+^ T cells from naïve and infected WT and WSX-1^−/−^ mice. (B) The frequencies of splenic Th1 effector CD4^+^ T cells derived from naïve and infected WT and WSX-1^−/−^ mice expressing Annexin V. (C) The mean fluorescence intensity of Annexin V expression by splenic Th1 effector CD4^+^ T cells from naïve and infected WT and WSX-1^−/−^ mice. (D) Representative histograms showing the levels of expression of Bcl-2 in naïve cells (CD44^−^ CD62L^+^, solid histograms) and Th1 effector CD4^+^ T cells (empty histograms) derived from naïve and infected WT (grey line) and WSX-1^−/−^ mice (black line). The results are the mean +/− SEM of the group with 3–5 mice per group. The results are representative of 2 independent experiments. * P<0.05 between WT and WSX-1^−/−^ mice.
(TIF)

Figure S5 **KLRG-1^+^Th1 cells that develop in malaria-infected WSX-1^−/−^ mice appear to be atypical terminally differentiated Th1 cells.** WT and WSX-1^−/−^ mice were infected with *P. berghei* NK65. (A) Representative plots showing KLRG-1 expression versus BrdU incorporation in splenic Th1 effector CD4^+^ T cells from naïve and infected WT and WSX-1−/− mice. (B) Gating strategy to define KLRG-1^+^ and KLRG-1^−^ effector T-bet^+^ CD4^+^ T cells. (C) Representative plots of IFN-γ versus TNF production within subdivided splenic KLRG-1^+^ and KLRG-1^−^ Th1 effector CD4^+^ T cell populations derived from naïve and infected WSX-1^−/−^ mice following in vitro PMA + ionomycin stimulation (D) The frequencies of polyfunctional CD4^+^ effector Th1 cells expressing IFN-γ and TNF within the KLRG-1^+^ and KLRG-1^−^ populations shown in B. The results are the mean +/− SEM of the group with 3–5 mice per group. The results are representative of 3 independent experiments. * P<0.05 between WT and WSX-1^−/−^ mice.
(TIF)

Figure S6 **Phenotypic profiling of CD4^+^T-bet^+^ KLRG-1^+^ and KLRG-1^−^ cells in WSX-1^−/−^ mice.** WT and WSX-1^−/−^ mice were infected i.v. with 10^4^ *P. berghei* NK65 pRBC. Expression of cytokine receptors and regulatory receptors by KLRG-1^+^ (black histograms) and KLRG-1^−^ (grey histograms) splenic Th1 effector CD4^+^ T cells from WSX-1−/− mice on days 9 and 14 of infection. Numbers show the mean fluorescence intensity of receptor expression for each KLRG population.
(TIF)

Figure S7 **Depletion of macrophage and dendritic cell populations attenuates IL-12 production and reduces Th1 CD4^+^ T cell terminal differentiation in infected WSX-1^−/−^ mice.** (A) Expression of IL-12p35 by different innate cell populations in the spleen of *P. berghei* NK65 infected WSX-1^−/−^ mice (day 13 p.i.), expressed relative to level of IL-12p35 gene expression by corresponding cells from infected WT mice. All APC populations were gated from CD3^−^, MHC II^+^ cells. Macrophages were sorted as CD11c^−^ F4-80^+^ cells, CD8^+^ and CD8^−^ DCs were gated from CD11c^+^ cells and the remaining MHC-II^+^APCs as CD11c^−^ F4-80^−^. (B, C) Absolute numbers of (B) splenic DCs and (C) macrophages in naïve and infected (Day 13 p.i.) WT and WSX-1^−/−^ mice. (D) Histogram plots showing the depletion of DC (CD11c^+^) and macrophage (F4-80^+^) cell populations in infected (day 13 p.i.) WSX-1^−/−^ mice following clodronate liposome administration from day 7 of infection. (E) The plasma levels of IL-12p70 in naïve, day 13 infected WT and WSX-1^−/−^ and day 13 infected + clodronate liposome treated mice, as measured by cytokine bead array. (F, H) Representative histograms showing (F) T-bet expression by splenic CD4^+^ effector (CD44^+^ CD62L^−^) T cells and (H) KLRG-1 expression by CD4^+^ effector T-bet^+^ T cells from infected (day 13 p.i.) control and clodronate-liposome treated WT and WSX-1^−/−^ mice. (G, I) The total numbers of splenic CD4^+^ effector T cells expressing (G) T-bet and (I) CD4^+^ effector T-bet^+^ T cells expressing KLRG-1 in infected control and clodronate-treated WT and WSX-1^−/−^ mice. The results are the mean +/− SEM of the group with 3–4 mice per group. * P<0.05 between infected WT and infected WSX-1^−/−^. # P<0.05 between WSX-1^−/−^ clodronate treated and WSX-1^−/−^ control treated mice.
(TIF)

Figure S8 **Depletion of Foxp3^+^ regulatory T cells does not lead to aberrant Th1 responses during malaria infection.** DEREG mice and littermate control mice were infected i.v. with 10^4^ *P. berghei* NK65 pRBC. Mice were treated every second day with 200 ng diphtheria toxin (DT), starting one day prior to infection. (A) The expression of Foxp3-GFP by CD4^+^ T cells from DEREG mice immediately prior to DT administration and on day 13 of infection. (B) The expression of Foxp3, as detected by intracellular staining, on CD4^+^ T cells from DT treated DEREG and littermate mice on day 13 of infection. (C) The expression of T-bet by CD4^+^CD44^+^CD62L^−^ cells from DT Treated DEREG and littermate mice on day 13 of infection. (D) The expression of KLRG-1 by CD4^+^CD44^+^CD62L^−^T-bet^+^ cells from DT Treated DEREG and littermate mice on day 13 of infection.
(TIF)

Figure S9 **Validation of multiparameter flow-cytometry staining panels by FMO and isotype control staining.** Splenocytes from naïve, day 13 WT and WSX-1^−/−^ infected mice were surface stained for different markers, permeabilized with Foxp3 fixation/permeabilization buffers followed by intracellular staining of FoxP3 or T-bet. For cytokine control staining, splenocytes were incubated for 5 h in the presence of PMA, ionomycin and Brefeldin A, followed by the staining protocol described in Materials and Methods. Staining controls (FMO staining with the addition of the corresponding isotype control antibody) are shown in histogram overlay plots for the corresponding CD4^ +^ T cell population used for gating.
(TIF)
