# Supplementary figures and images for: IL-27 Receptor Signalling Restricts the Formation of Pathogenic, Terminally Differentiated Th1 Cells during Malaria Infection by Repressing IL-12 Dependent Signals
Source: PLoS Pathog. 2013 Apr 11;9(4):e1003293. doi: 10.1371/journal.ppat.1003293 (PMC3623720; doi:10.1371/journal.ppat.1003293)

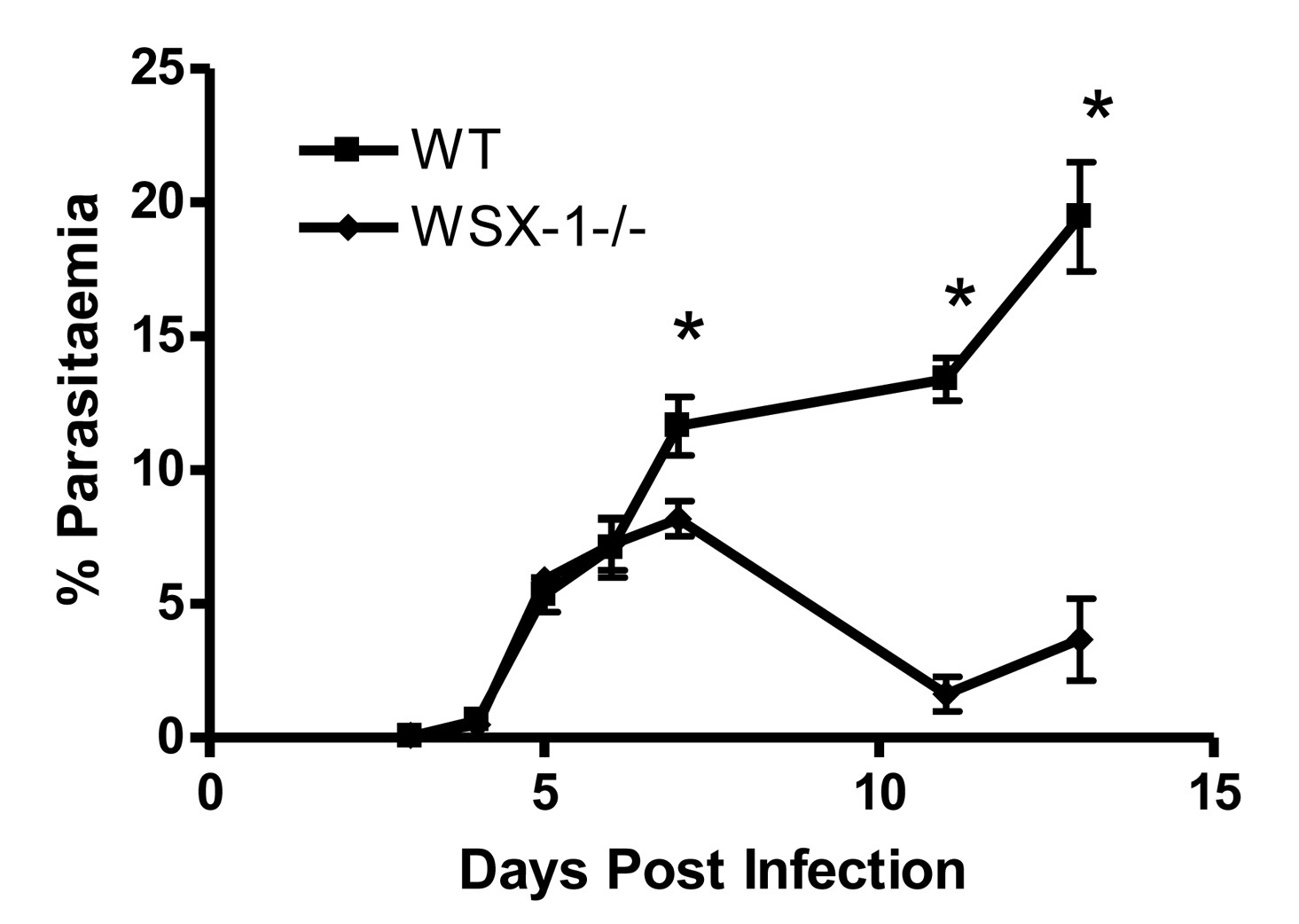

Supplement: Figure S1 — The course of P. berghei NK65 infection in WT and WSX-1−/− mice. WT and WSX-1−/− mice were infected i.v. with 104 P. berghei NK65 pRBC. The peripheral parasite burdens in WT and WSX-1−/− mice were assessed on thin smears by microscopy. * P<0.05 between WT and WSX-1−/− mice. (TIF) [file ppat.1003293.s001.tif]

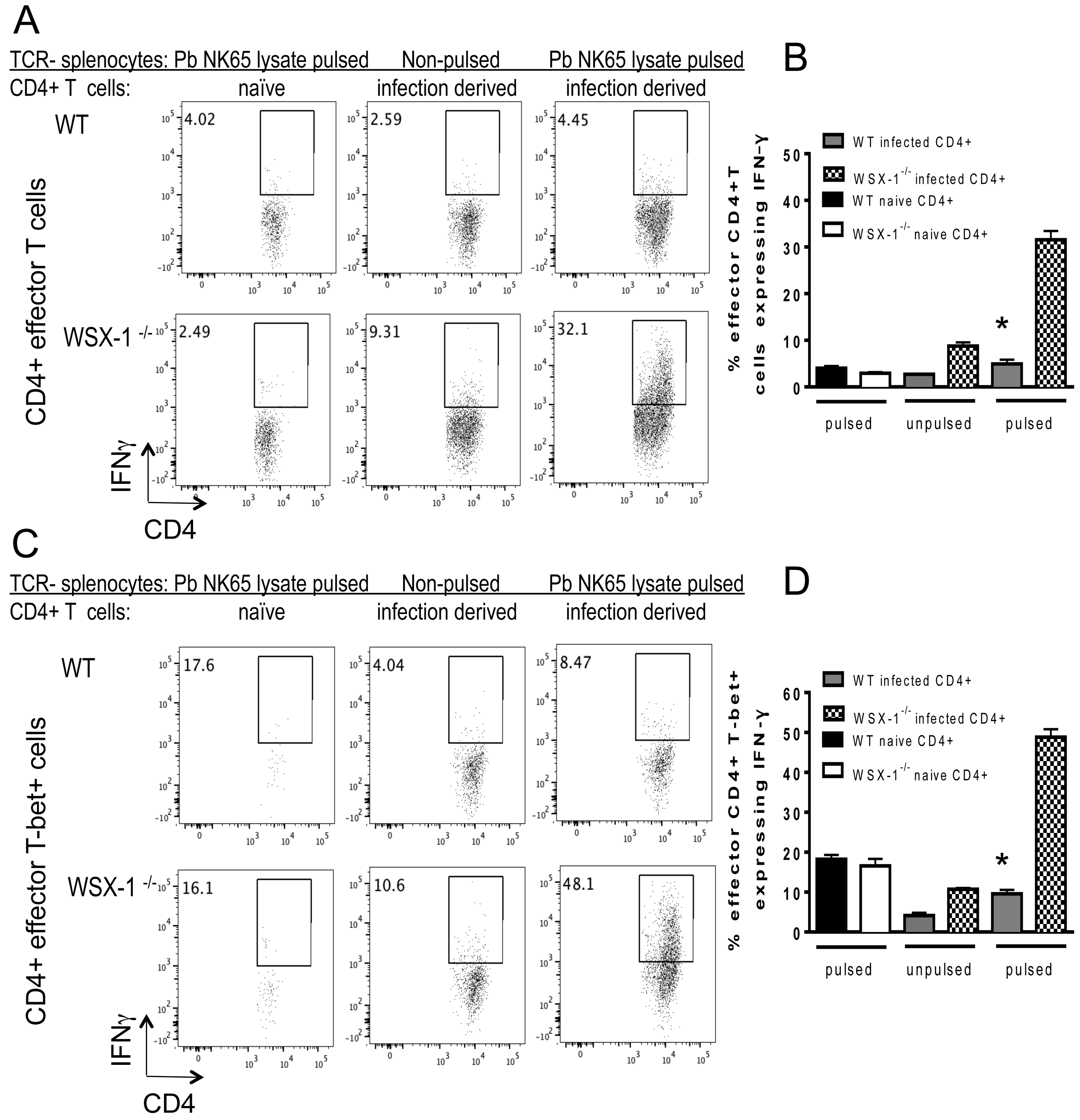

Supplement: Figure S2 — Malaria specific CD4+ T cells from infected WSX-1−/− mice produce significantly more IFN-γ than corresponding cells from infected WT mice. Splenic CD4+ T cells were purified from naïve and malaria-infected (day 13 p.i.) WT and WSX-1−/− infected mice and were restimulated in vitro with P. berghei NK65 pulsed APCs obtained from naïve mice. Representative plots showing IFN-γ expression by CD4+ effector T cells (A) or Th1 effector CD4+ T cells (C) after overnight restimulation. The frequencies of CD4+ effector T cells (B) or Th1 effector CD4+ T cells (D) expressing IFN-γ from WT and WSX-1−/− naïve or infection-derived CD4+ T cells. The results are the mean +/− SEM of 3 independent wells. * P<0.05 between WT and WSX-1−/− pulsed groups. (TIF) [file ppat.1003293.s002.tif]

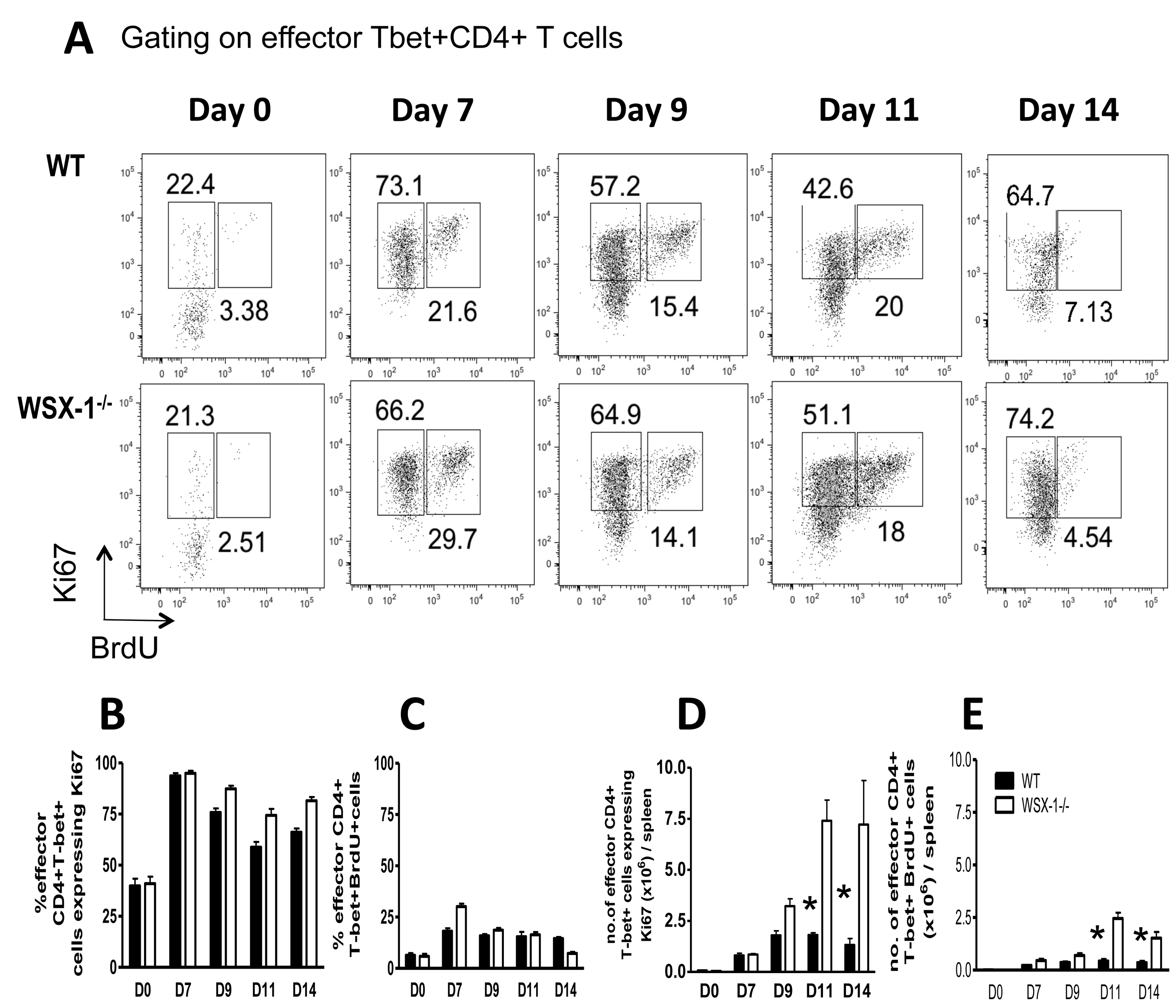

Supplement: Figure S3 — Restriction of splenic Th1 response in WT mice during malaria infection is not due to impaired Th1 cell proliferation. WT and WSX-1−/− mice were infected i.v. with 104 P. berghei NK65 pRBC. 1.25 mg of BrdU was injected i.p. 1 h before animals were culled. (A) Representative plots showing Ki67 expression versus BrdU incorporation by splenic Th1 effector CD4+ T cells from naïve and infected WT and WSX-1−/− mice. Numbers within plots represent the frequencies of Ki67+ BrdU- cells (top left) and Ki67+ BrdU+ (bottom right). (B–E) The frequencies (B–C) and total numbers (D–E) of splenic CD4+ effector T-bet+ T cells expressing (B, D) Ki67 and (C, E) incorporating BrdU. The results are the mean +/− SEM of the group with 3–5 mice per group. The results are representative of 3 independent experiments. * P<0.05 between WT and WSX-1−/− mice. (TIF) [file ppat.1003293.s003.tif]

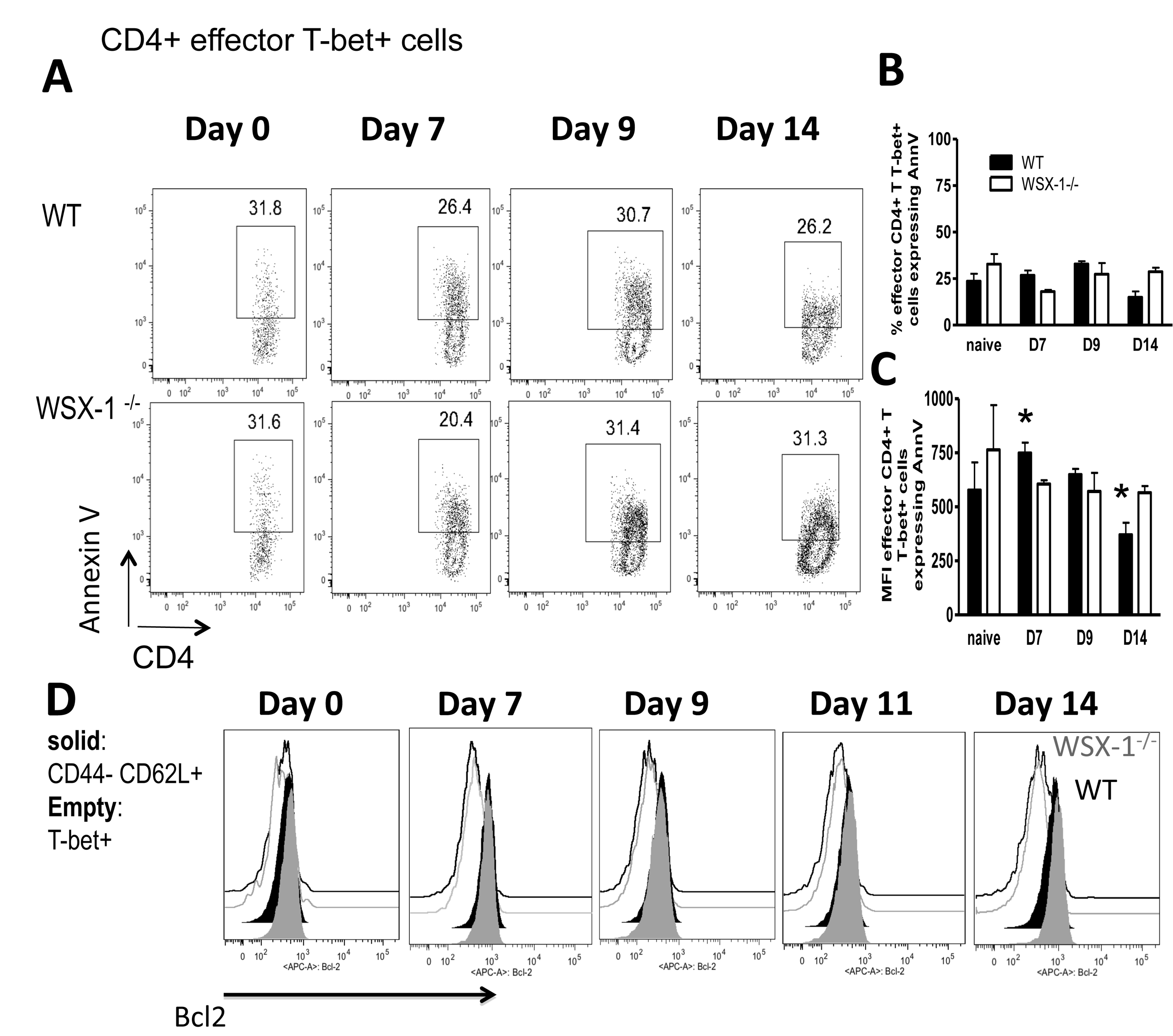

Supplement: Figure S4 — Restriction of splenic Th1 response in WT mice is not due to IL-27R- direct or indirect promotion of Th1 cell apoptosis or altered survival. WT and WSX-1−/− mice were infected i.v. with 104 P. berghei NK65 pRBC. (A) Representative plots showing Annexin V expression by splenic Th1 effector CD4+ T cells from naïve and infected WT and WSX-1−/− mice. (B) The frequencies of splenic Th1 effector CD4+ T cells derived from naïve and infected WT and WSX-1−/− mice expressing Annexin V. (C) The mean fluorescence intensity of Annexin V expression by splenic Th1 effector CD4+ T cells from naïve and infected WT and WSX-1−/− mice. (D) Representative histograms showing the levels of expression of Bcl-2 in naïve cells (CD44− CD62L+, solid histograms) and Th1 effector CD4+ T cells (empty histograms) derived from naïve and infected WT (grey line) and WSX-1−/− mice (black line). The results are the mean +/− SEM of the group with 3–5 mice per group. The results are representative of 2 independent experiments. * P<0.05 between WT and WSX-1−/− mice. (TIF) [file ppat.1003293.s004.tif]

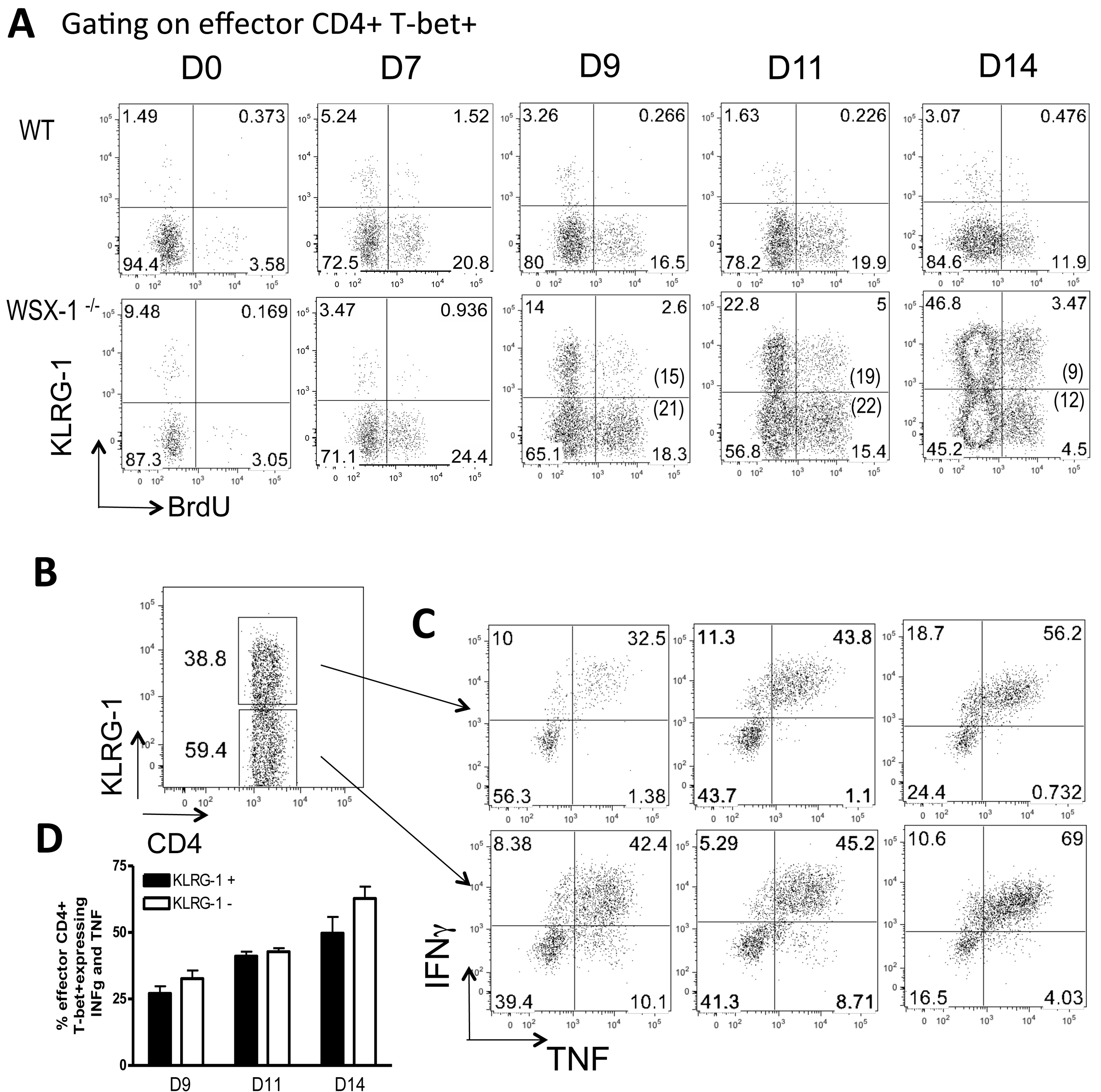

Supplement: Figure S5 — KLRG-1+Th1 cells that develop in malaria-infected WSX-1−/− mice appear to be atypical terminally differentiated Th1 cells. WT and WSX-1−/− mice were infected with P. berghei NK65. (A) Representative plots showing KLRG-1 expression versus BrdU incorporation in splenic Th1 effector CD4+ T cells from naïve and infected WT and WSX-1−/− mice. (B) Gating strategy to define KLRG-1+ and KLRG-1− effector T-bet+ CD4+ T cells. (C) Representative plots of IFN-γ versus TNF production within subdivided splenic KLRG-1+ and KLRG-1− Th1 effector CD4+ T cell populations derived from naïve and infected WSX-1−/− mice following in vitro PMA + ionomycin stimulation (D) The frequencies of polyfunctional CD4+ effector Th1 cells expressing IFN-γ and TNF within the KLRG-1+ and KLRG-1− populations shown in B. The results are the mean +/− SEM of the group with 3–5 mice per group. The results are representative of 3 independent experiments. * P<0.05 between WT and WSX-1−/− mice. (TIF) [file ppat.1003293.s005.tif]

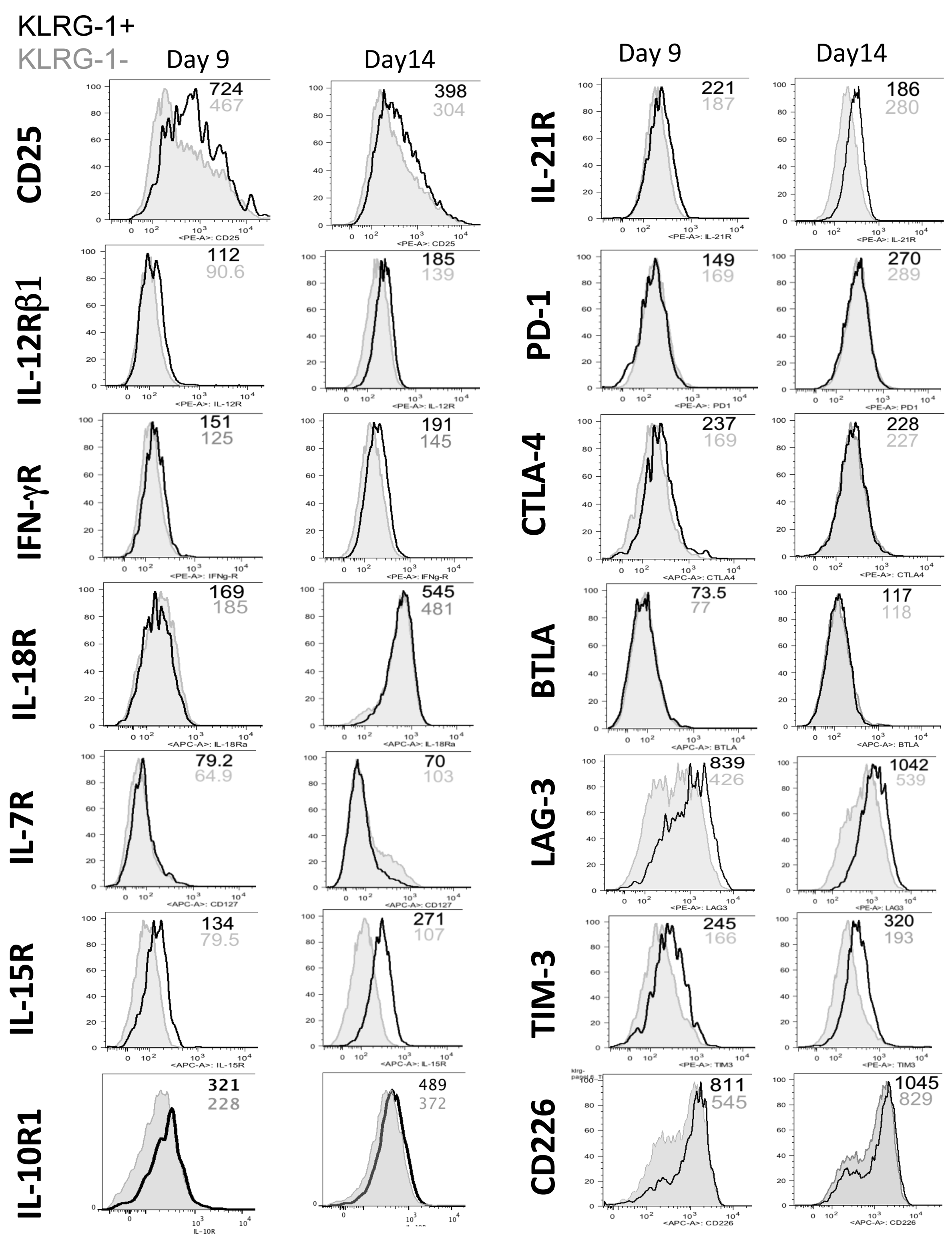

Supplement: Figure S6 — Phenotypic profiling of CD4+T-bet+ KLRG-1+ and KLRG-1− cells in WSX-1−/− mice. WT and WSX-1−/− mice were infected i.v. with 104 P. berghei NK65 pRBC. Expression of cytokine receptors and regulatory receptors by KLRG-1+ (black histograms) and KLRG-1− (grey histograms) splenic Th1 effector CD4+ T cells from WSX-1−/− mice on days 9 and 14 of infection. Numbers show the mean fluorescence intensity of receptor expression for each KLRG population. (TIF) [file ppat.1003293.s006.tif]

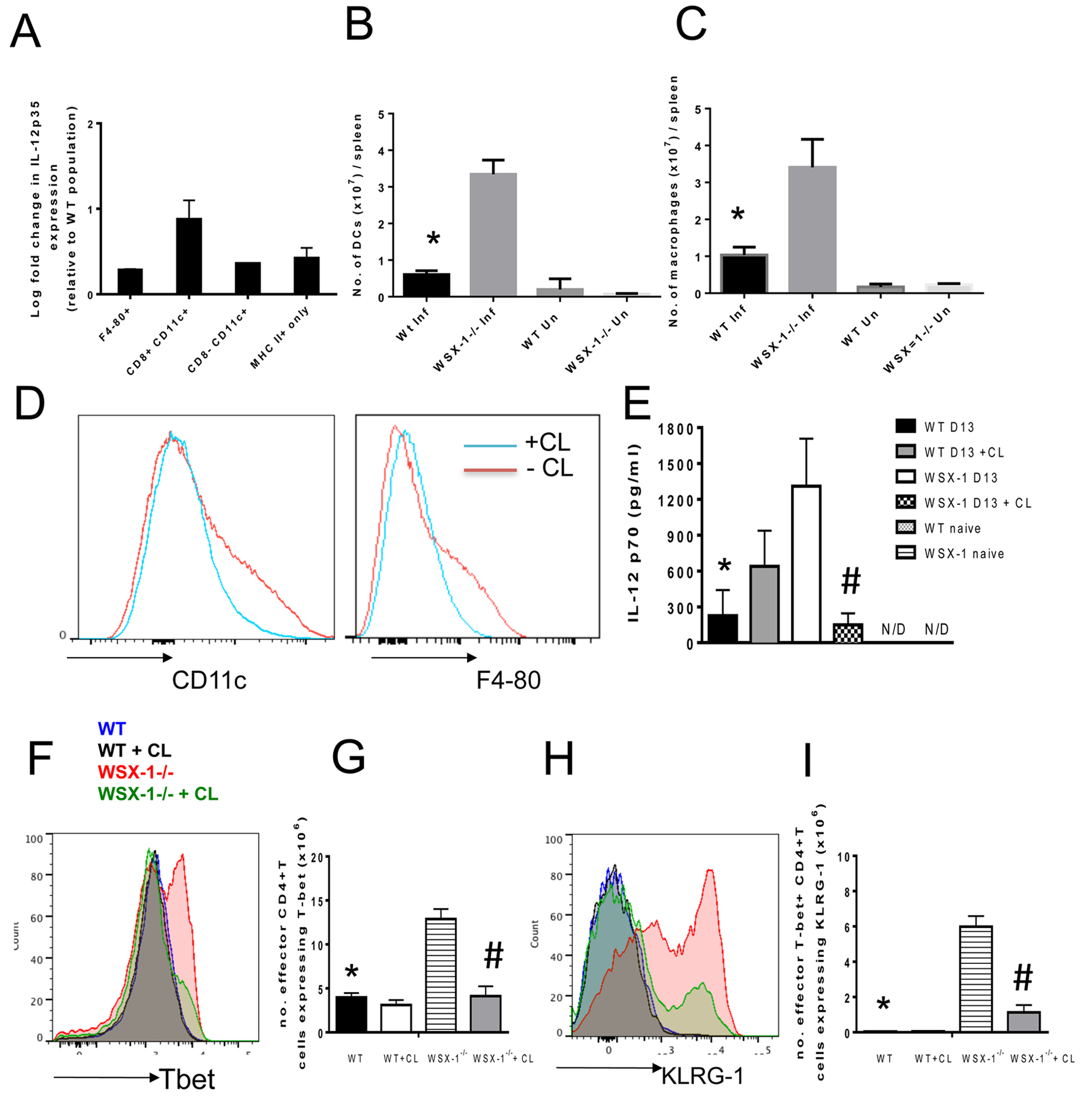

Supplement: Figure S7 — Depletion of macrophage and dendritic cell populations attenuates IL-12 production and reduces Th1 CD4+ T cell terminal differentiation in infected WSX-1−/− mice. (A) Expression of IL-12p35 by different innate cell populations in the spleen of P. berghei NK65 infected WSX-1−/− mice (day 13 p.i.), expressed relative to level of IL-12p35 gene expression by corresponding cells from infected WT mice. All APC populations were gated from CD3−, MHC II+ cells. Macrophages were sorted as CD11c− F4-80+ cells, CD8+ and CD8− DCs were gated from CD11c+ cells and the remaining MHC-II+APCs as CD11c− F4-80−. (B, C) Absolute numbers of (B) splenic DCs and (C) macrophages in naïve and infected (Day 13 p.i.) WT and WSX-1−/− mice. (D) Histogram plots showing the depletion of DC (CD11c+) and macrophage (F4-80+) cell populations in infected (day 13 p.i.) WSX-1−/− mice following clodronate liposome administration from day 7 of infection. (E) The plasma levels of IL-12p70 in naïve, day 13 infected WT and WSX-1−/− and day 13 infected + clodronate liposome treated mice, as measured by cytokine bead array. (F, H) Representative histograms showing (F) T-bet expression by splenic CD4+ effector (CD44+ CD62L−) T cells and (H) KLRG-1 expression by CD4+ effector T-bet+ T cells from infected (day 13 p.i.) control and clodronate-liposome treated WT and WSX-1−/− mice. (G, I) The total numbers of splenic CD4+ effector T cells expressing (G) T-bet and (I) CD4+ effector T-bet+ T cells expressing KLRG-1 in infected control and clodronate-treated WT and WSX-1−/− mice. The results are the mean +/− SEM of the group with 3–4 mice per group. * P<0.05 between infected WT and infected WSX-1−/−. # P<0.05 between WSX-1−/− clodronate treated and WSX-1−/− control treated mice. (TIF) [file ppat.1003293.s007.tif]

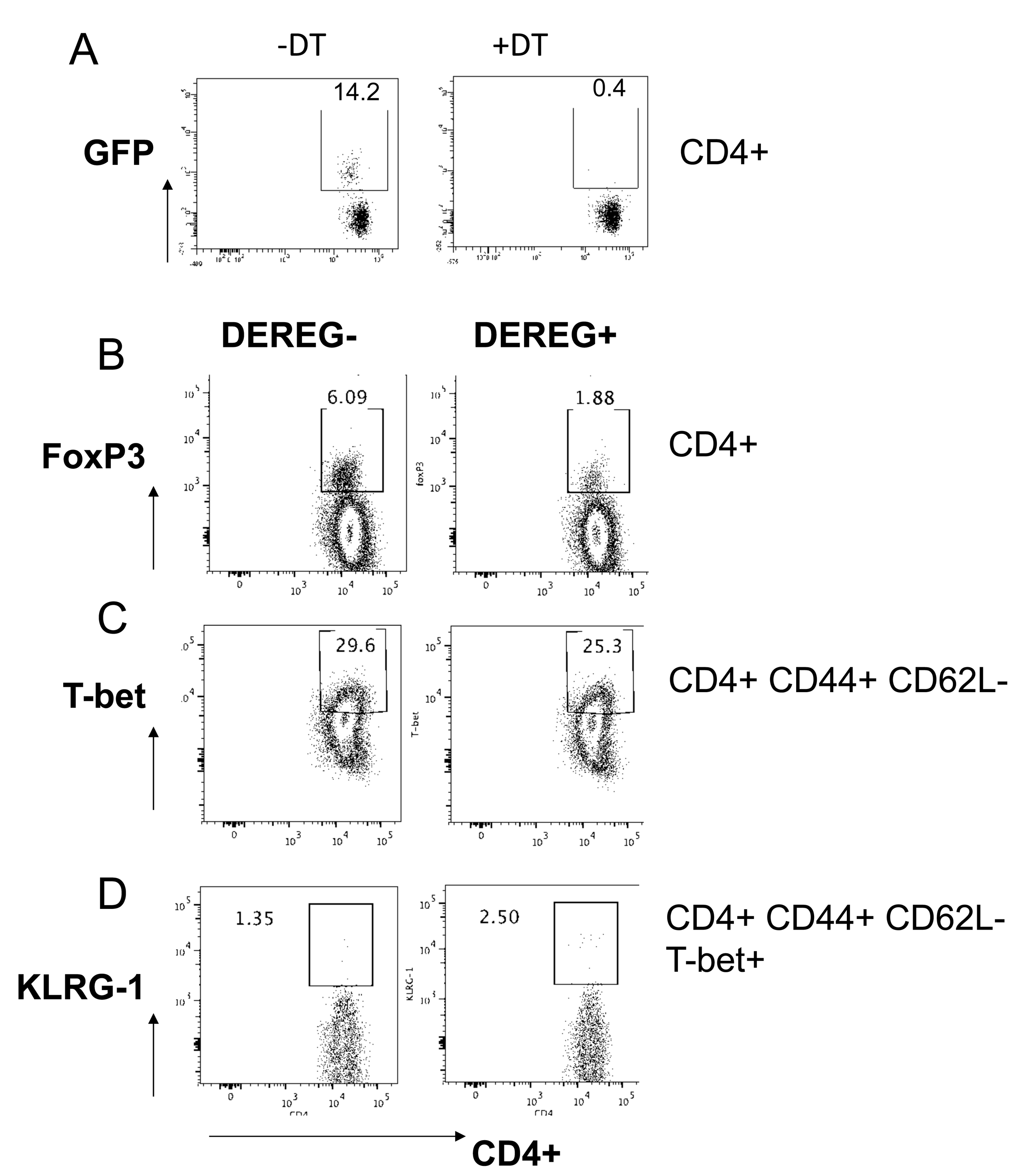

Supplement: Figure S8 — Depletion of Foxp3+ regulatory T cells does not lead to aberrant Th1 responses during malaria infection. DEREG mice and littermate control mice were infected i.v. with 104 P. berghei NK65 pRBC. Mice were treated every second day with 200 ng diphtheria toxin (DT), starting one day prior to infection. (A) The expression of Foxp3-GFP by CD4+ T cells from DEREG mice immediately prior to DT administration and on day 13 of infection. (B) The expression of Foxp3, as detected by intracellular staining, on CD4+ T cells from DT treated DEREG and littermate mice on day 13 of infection. (C) The expression of T-bet by CD4+CD44+CD62L− cells from DT Treated DEREG and littermate mice on day 13 of infection. (D) The expression of KLRG-1 by CD4+CD44+CD62L−T-bet+ cells from DT Treated DEREG and littermate mice on day 13 of infection. (TIF) [file ppat.1003293.s008.tif]

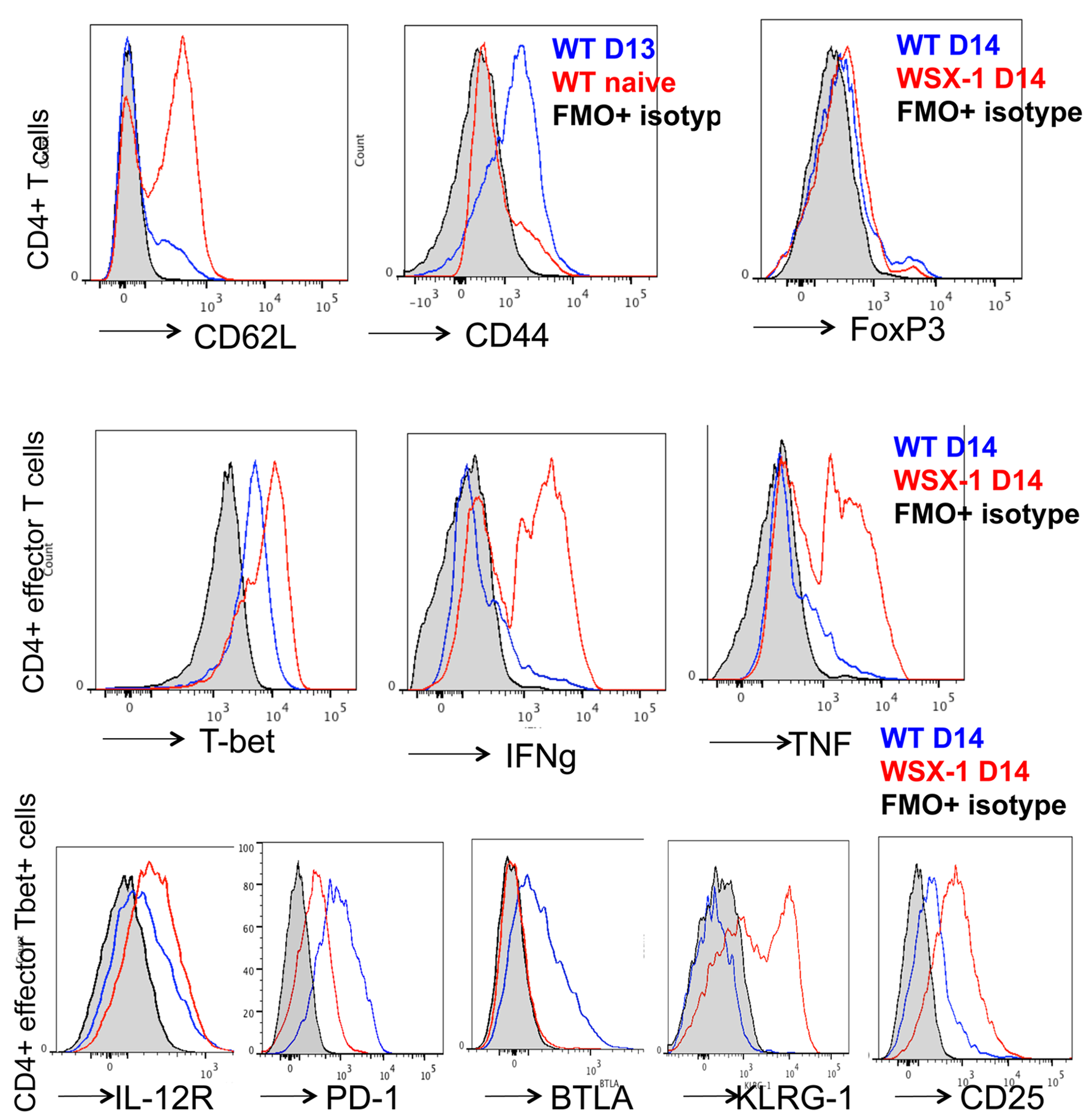

Supplement: Figure S9 — Validation of multiparameter flow-cytometry staining panels by FMO and isotype control staining. Splenocytes from naïve, day 13 WT and WSX-1−/− infected mice were surface stained for different markers, permeabilized with Foxp3 fixation/permeabilization buffers followed by intracellular staining of FoxP3 or T-bet. For cytokine control staining, splenocytes were incubated for 5 h in the presence of PMA, ionomycin and Brefeldin A, followed by the staining protocol described in Materials and Methods. Staining controls (FMO staining with the addition of the corresponding isotype control antibody) are shown in histogram overlay plots for the corresponding CD4 + T cell population used for gating. (TIF) [file ppat.1003293.s009.tif]
